# CdTe Quantum Dots Encapsulated on Perovskite Grains Enable Highly Efficient and Stable Perovskite Solar Cells

**DOI:** 10.1002/adma.202521104

**Published:** 2026-01-10

**Authors:** Wenhao Zhao, Deyou Lin, Riming Sun, Zhiyu Fang, Pengfei Guo, Yadong Xu, Hongqiang Wang, Feng Yan

**Affiliations:** ^1^ State Key Laboratory of Solidification Processing Center for Nano Energy Materials School of Materials Science and Engineering Shaanxi Laboratory for Advanced Materials Northwestern Polytechnical University and Shaanxi Joint Laboratory of Graphene (NPU) Xi'an P. R. China; ^2^ Department of Applied Physics Research Centre for Organic Electronics The Hong Kong Polytechnic University Kowloon Hong Kong P. R. China; ^3^ Research & Development Institute of Northwestern Polytechnical University in Shenzhen Shenzhen P. R. China

**Keywords:** CdTe quantum dots, microscale carrier management, microscopic p‐n heterointerfaces, microstructural integrity, perovskite solar cells

## Abstract

Solution‐processed polycrystalline perovskites are inevitably endowed with inherent discontinuity at device heterointerfaces, which creates numerous interface segments that demand deliberate engineering of metastable interfacial configurations. Nevertheless, critical challenge remains in synchronously manipulating interfacial microscale carrier management while maintaining their microstructural integrity under operational stresses. Herein we demonstrate a strategy to fabricate localized microscopic p‐n heterointerfaces with high coherence and ionic bridging through encapsulating well‐defined p‐type CdTe quantum dots (QDs) on n‐type perovskite grains. Surface embeddings of such QDs establish unidirectionally aligned built‐in electric fields that facilitate directional carrier transport across micro‐heterointerfaces while expanding depletion regions to minimize recombination loss. Moreover, CdTe‐induced heteroepitaxial growth yields dislocation‐less interfaces between CdTe and perovskite, simultaneously passivating accessible defects of iodine vacancies and undercoordinated Pb^2+^ at both the surface and grain boundaries, enabling high‐crystallinity perovskite films with robust microstructures. Given these striking merits, a record‐high efficiency of 26.73% (certified 26.02%) with a remarkable open‐circuit voltage of 1.222 V is achieved, setting a new performance benchmark among regular perovskite solar cells, along with pronounced operational stability with negligible efficiency degradation after nearly 700 h. This work pioneers a transformative laser‐mediated microscopic heterointerface engineering strategy that fundamentally reengineers microstructural carrier management and long‐term durability in advanced optoelectronics.

## Graphical abstract

Present work demonstrates an efficient laser‐assisted localized interfacial engineering strategy that achieves epitaxial‐grade atomic coherence in spatially defined p‐n microscopic heterointerfaces, via precisely embedding p‐type CdTe QDs into an n‐type perovskite matrix, which concurrently establishes dual optimization of microscale carrier management and microstructural integrity, consequently resulting in a new performance benchmark of 26.73% in regular PSCs.

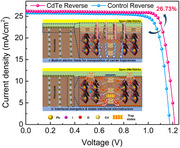

## 1 Introduction

The past decade has witnessed unprecedented progress in perovskite solar cells (PSCs), with single‐junction configuration so far achieving certified power conversion efficiencies (PCEs) exceeding 27% while maintaining their advantages in solution processability and manufacturing scalability [1, 2, 3]. Nevertheless, solution‐processed polycrystalline perovskites exhibit inherent discontinuity at device heterointerfaces, comprising numerous interfacial segments that demand deliberate engineering of metastable interfacial configurations [4, 5]. The primary obstacle lies in simultaneously manipulating charge carrier trajectories of each individual interfacial microscopic region to enhance transport integration while preserving their microstructural integrity under operational stresses [4, 5, 6, 7]. This delicate balance requires atomic‐ or molecular‐scale precision modulation over interfacial energetics while withstanding the combined effects of moisture, thermal, and light degradation pathways on susceptible interface microstructure [8, 9]. Recent advances have suggested that heterojunction engineering within or interfacial perovskite can intentionally orient transport of charge carrier by introducing extra built‐in electric fields (BEFs) or graded energy funnels, playing a dominating role in carrier management of PSCs [6, 7, 10, 11, 12, 13]. Several state‐of‐the‐art strategies in terms of 2D/3D hybrids or 3D/3D bilayer stacks [14, 15, 16], phase heterojunctions [12, 17], and p‐n junctions [18, 19], have demonstrated remarkable success in collectively addressing critical trade‐offs among charge transport, defect tolerance, and phase homogeneity. Therefore, constructing high‐quality heterojunctions has become significantly imperative for developing high‐efficiency and stable PSCs.

Despite these advances, fundamental limitations in most conventional heterostructures stem from the lack of precise modulation over heterojunction morphology and electronic structure at the microscale, leading to lattice‐mismatch‐induced disordered interfaces [20], undesirable energy disorder [16], and insufficient BEFs [21]. These cumulative effects  would arouse substantial recombination and carrier transport loss, less‐than‐ideal efficiency, and environmental instability of PSCs [9]. Innovative microscopic heterointerfaces, by virtue of their enlarged surface area, could maximize interfacial interactions through increased coupling sites, which partly compensates for  lattice constraints and can be further strengthened by high lattice matching [22, 23]. Additionally, the microscale dimensions allow for fine‐tuned band energetics that creates substantial BEFs for rapid carrier separation and transport while minimizing energy offsets for reduced non‐radiative recombination losses [24]. Leveraging the inherent merits of microscopic heterointerfaces, it thus is significantly promising to simultaneously achieve deterministic carrier trajectory manipulation via microscale energetics landscapes and microstructural integrity preservation for highly efficient and stable PSCs, in which such a dual requirement has been rarely considered.

**SCHEME 1:**
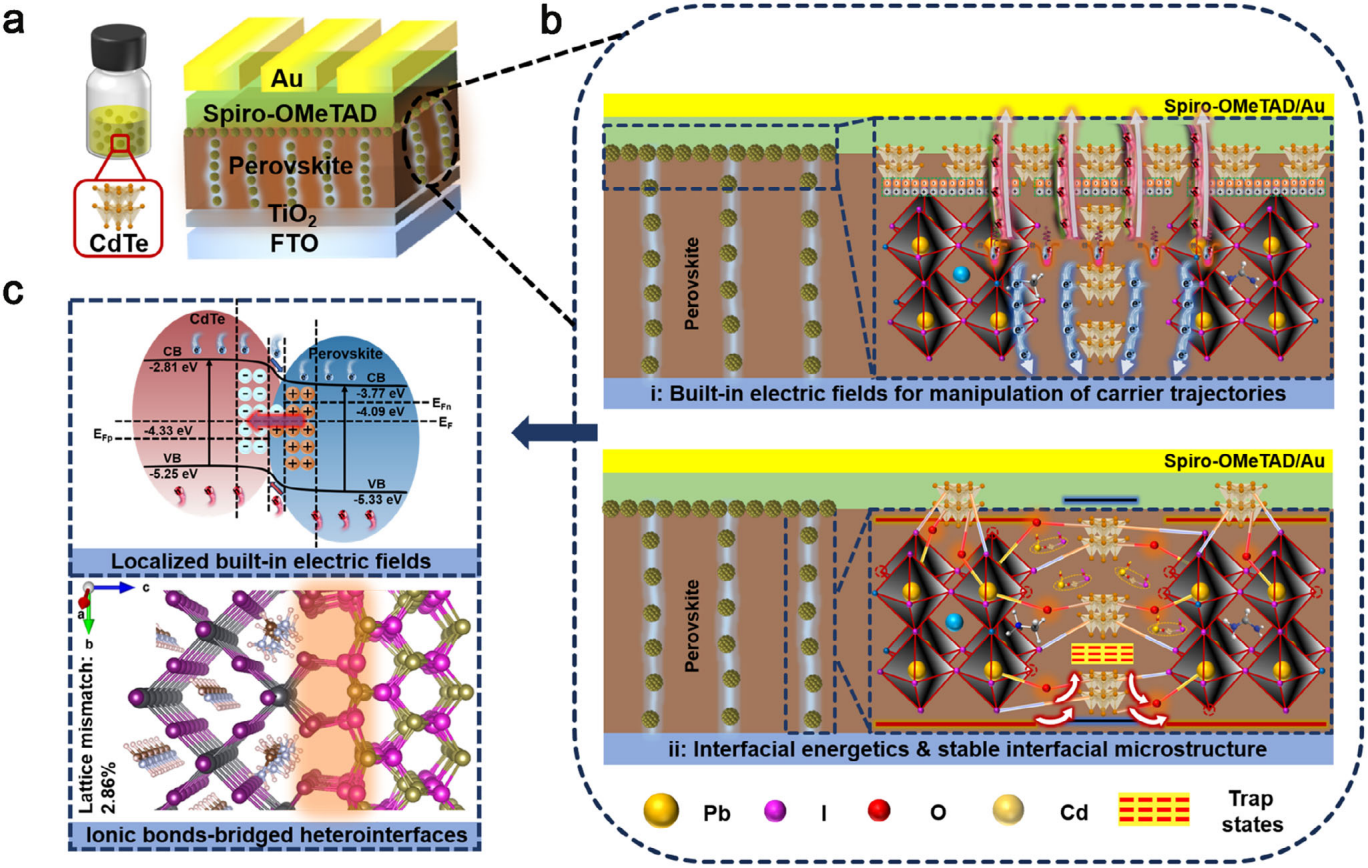
a) Schematic illustration of configuration of PSCs with embedded CdTe QDs. Schematic illustration of b) the effect of p‐n microscopic heterointerfaces on carrier dynamics regulation, energy level alignment, and defect manipulation of the perovskite, as well as c) ionic bonds‐bridged heterointerfaces with localized built‐in electric fields.

In this work, we pioneer a localized interfacial engineering strategy that achieves both high coherence and robust ionic bridging in microscopic p‐n heterointerfaces, which is significantly challenging for conventional colloid synthesis with ligand exchange [25], via precisely embedding well‐defined p‐type CdTe quantum dots (QDs) on n‐type perovskite grains. This concurrently establishes dual optimizations of microscale carrier management and microstructural integrity for record‐breaking PSCs. By elaborately modulating the concentration of embedded p‐type CdTe QDs, accurate spatial positioning and homogeneous distribution throughout the perovskite matrix are achieved (Scheme 1a). The CdTe QDs located on the surface establish unidirectionally aligned BEFs with perovskite that significantly enhance field strength to facilitate directional charge transport across the heterointerface while expanding depletion region that minimizes recombination loss (Scheme 1b,c top). Moreover, the incorporated CdTe serves as passivation agents that mitigate dual defects of iodine vacancies and undercoordinated Pb^2^^+^ at both surface and grain boundaries (GBs) to reduce the depth of potential wells for holes, while their near‐perfect lattice matching with perovskite facilitates dislocation‐less interfacial growth. This collectively enables minimal trap states and robust durability of microstructures in PSCs (Scheme 1b,c bottom). Benefiting from these desirable merits, a target PSC delivers a top record PCE of 26.73% (certified 26.02%) with an outstanding open‐circuit voltage (*V*_OC_) of 1.222 V at 1.56 eV bandgap, which ranks among the best‐performing regular PSCs. Furthermore, the device demonstrates pronounced environmental stability, maintaining stable shelf storage over 2000 h (RH of 60%), and over 98% of its initial efficiency under continuous maximum power point (MPP) tracking for nearly 700 h. The present study pioneers new avenues for manipulating carrier management via laser‐mediated microscopic heterointerface engineering, with significant implications for advanced photovoltaics and broader optoelectronic applications.

## 2 Result

### 2.1 CdTe QDs Embedded in Perovskite Films

**FIGURE 1:**
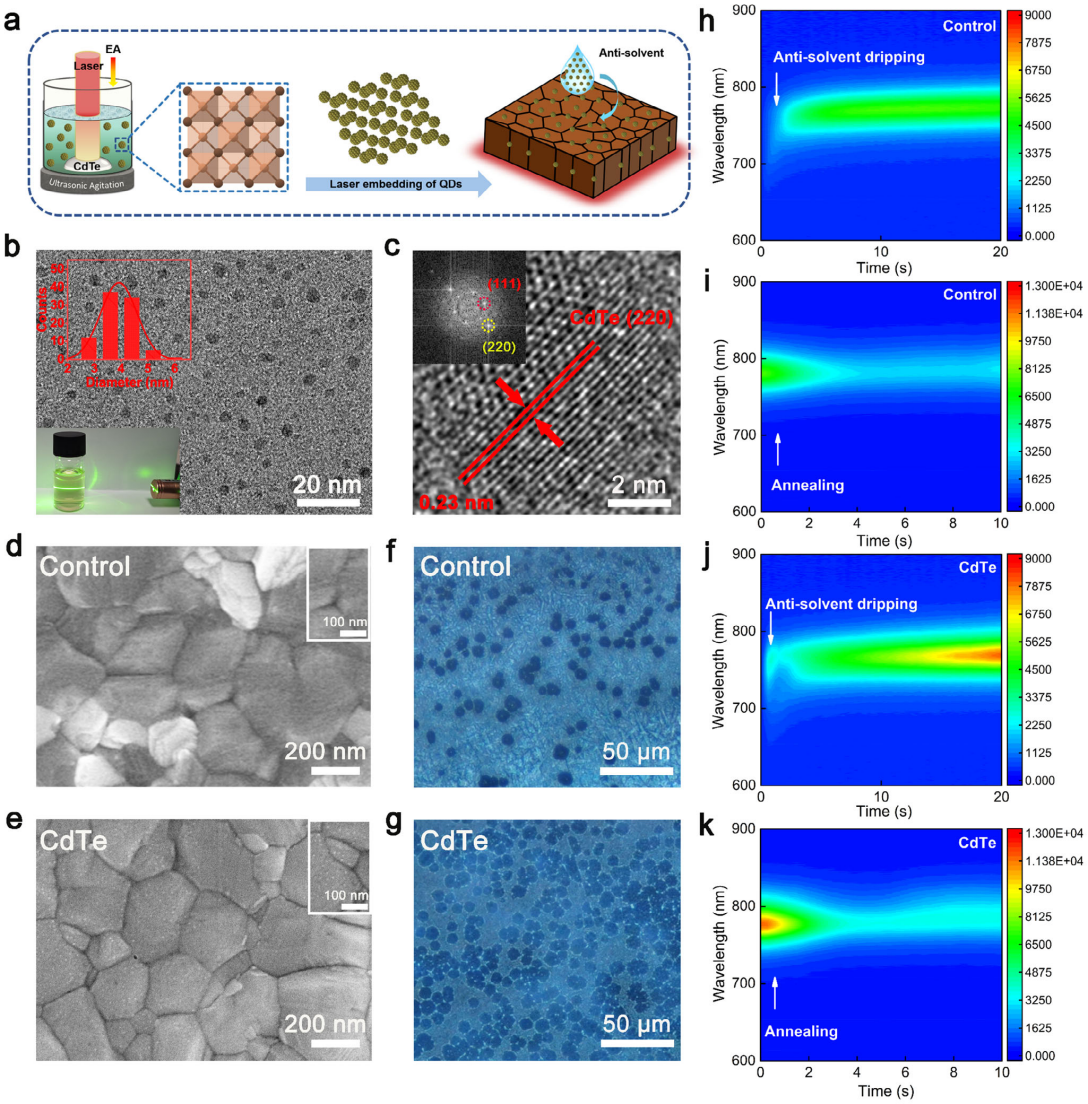
a) Schematic illustration of the laser process of CdTe QDs and subsequent embedding into perovskite film. b) TEM image of CdTe QDs (inset: size distribution diagram and Mie‐scattering image of colloids). c) HRTEM and corresponding FFT images of CdTe QDs. d,e) SEM images of the control and the target films. f,g) Optical microscopies of the perovskite precursor after dropping the anti‐solvent with and without CdTe QDs. In situ PL of h,i) the control and j,k) the target films.

As illustrated in Figure 1a, the technology of laser synthesis and processing of colloids (LSPC) was employed to generate well‐dispersed CdTe QDs with p‐type characteristics in the anti‐solvent, enabling their location at the surface and GBs of the perovskite film [26]. Subsequent to the optimization of laser fluence and concentration, stable p‐type CdTe colloids with an average diameter of 3.6 nm exhibiting Mie‐scattering were successfully synthesized (Figure 1b; Figure S1). High‐resolution transmission electron microscopy (HRTEM) image depicted in Figure 1c represents the lattice fringe spacing of 0.23 nm that corresponds precisely to the (220) plane of CdTe QDs. This is further evidenced by the fast Fourier transform (FFT), indicating both (111) and (220) planes. TEM‐energy dispersive spectroscopy (TEM‐EDS) analysis indicate a homogeneous distribution of all elements within the nanoparticles without elemental segregation during the laser irradiation, as illustrated in Figure S2. Furthermore, X‐ray photoelectron spectroscopy (XPS) suggests that the LSPC applied to CdTe do not significantly alter surface composition and chemical state of the QDs after laser irradiation (Figure S3).

The subsequent procedure for laser embedding of CdTe QDs into the mixed‐cation perovskite films (denoted as Cs_0.05_FA_0.85_MA_0.10_PbI_2.91_Br_0.09_) was conducted using a standard anti‐solvent method. These films are referred to as control and CdTe, respectively. Scanning electron microscopy (SEM) was conducted to evaluate the surface morphologies of different perovskite films. In comparison to the control, the CdTe film obviously displays well‐dispersed QDs on the surface with reduced PbI_2_ phase, which is mainly ascribed to faster nucleation that facilitates the transformation of PbI_2_ to PbI_3_^−^ and more uniform perovskite crystal growth (Figure 1d,e). This results in enhanced crystallinity of film with preferential grain growth along dominated planes (Figure S4) [27]. In addition, the XRD analysis reveals no significant phase changes or peak shifts, indicating the absence of CdTe within the perovskite lattice. To investigate the effect of CdTe on the nucleation and crystallization process of perovskite films, we utilized optical microscopy for further observation. This involved depositing the perovskite precursor onto a TiO_2_ substrate, followed by the addition of an anti‐solvent with and without CdTe QDs. It was found that the introduction of CdTe triggers the formation of a greater number of nucleation sites and enhances the nucleation rate compared to the control (Figure 1f,g). Further real‐time monitoring of the crystallization process of the perovskite film was achieved adopting in situ photoluminescence (PL) measurements. As shown in Figure 1h‐k, the PL intensity of CdTe film shows a shorter time to reach higher intensity and a greater increase in intensity in comparison with the control following the application of the antisolvent during spin coating and subsequent annealing process. This behavior suggests a higher nucleation rate and improved nucleation density in the CdTe film, agreeing well with the images of optical microscopy [28].

**FIGURE 2:**
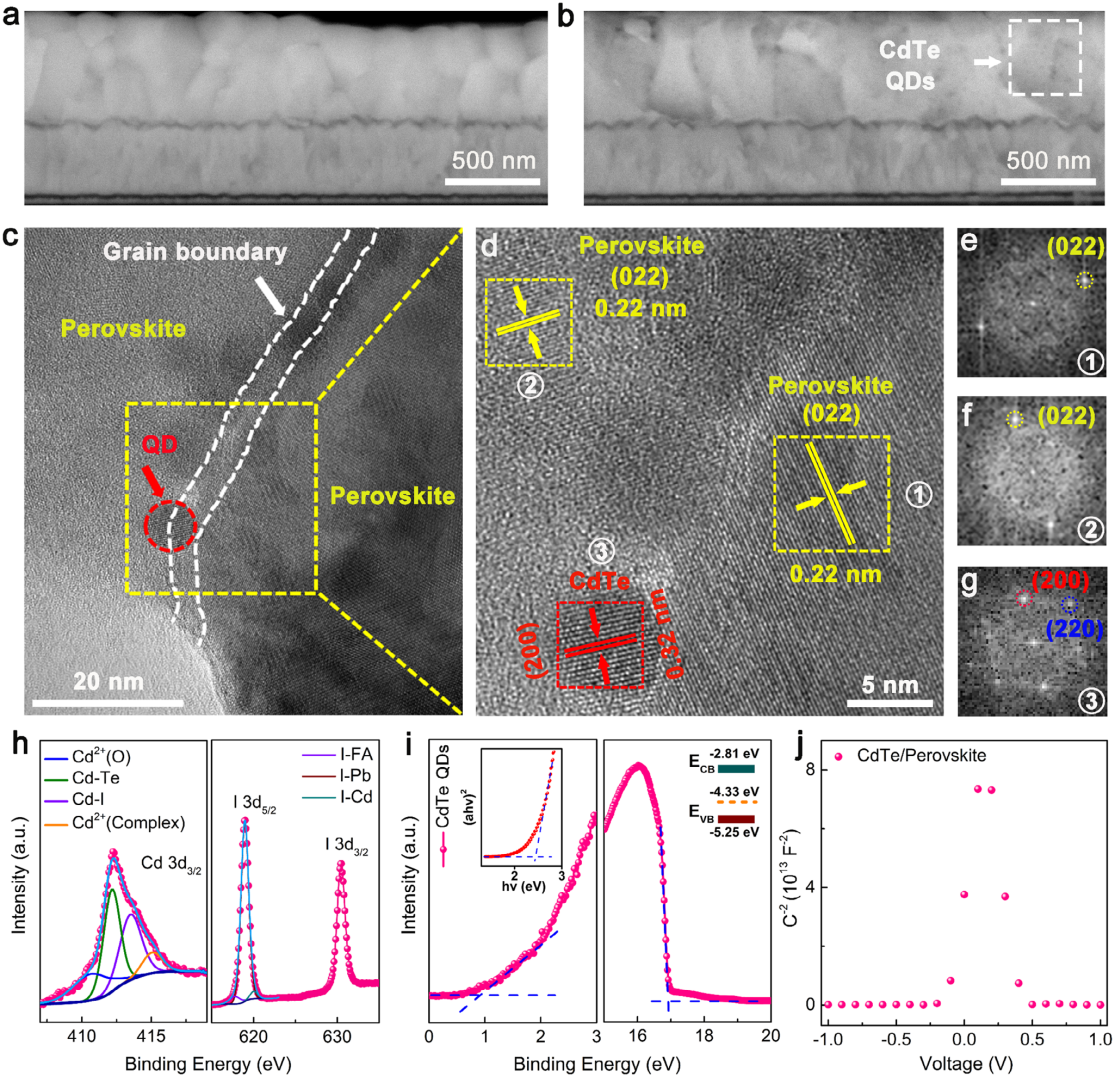
Cross‐sectional BSE images of a) control and b) CdTe films. c,d) HRTEM images (magnified yellow region) of perovskite film with laser embedding of CdTe QDs. FFT‐transformation images of e,f) the perovskite (labeled as yellow area) and g) the CdTe QDs (labeled as red area). h) High‐resolution XPS spectra of Cd 3d_3/2_ and I 3d_3/2_ for CdTe QDs embedded perovskite film. i) UPS (inset: Tauc plot and energy level diagram) of laser generated CdTe QDs. j) Mott‐Schottky plots of CdTe QDs embedded perovskite matrix.

In order to investigate the spatial distribution of CdTe QDs throughout the entire film thickness, cross‐sectional backscattered scanning electron (BSE) microscopy was used by leveraging the atomic number contrast, as reflected by the intensity of the backscattered electron signals [29]. As illustrated in Figure 2a,b, the presence of black dots in the CdTe film can be attributed to the lower atomic weights of Cd and Te compared to Pb, which are distributed throughout the entire layer, while the control film does not exhibit any significant contrast. Further evidence from the time‐of‐flight secondary‐ion mass spectroscopy (ToF‐SIMS) shows that the CdTe QDs are uniformly distributed in the perovskite matrix, and their content on the surface is higher than that in the bulk film (Figure S5). The HRTEM was further conducted to determine the location of CdTe QDs. Figure 2c illustrates the presence of CdTe QDs at the GBs of the perovskite film, providing compelling evidence of successful embedding. This is supported by the identification of characteristic lattice spacings of 0.22 and 0.32 nm, which correspond to the (022) plane of perovskite and the (200) plane of CdTe, respectively, as shown in Figure 2d. Further verification by the FFT analysis illustrated in Figure 2e–g, the result sequentially reveals the (022) plane of perovskite, along with the (200) and (220) planes of CdTe.

Furthermore, the XPS was employed to analyze the chemical state of CdTe in the film and to validate the chemical bonding between CdTe and perovskite. Figure 2h exhibits the high‐resolution XPS spectrum of Cd 3d_3/2_ and I 3d in the target perovskite film, where the Cd 3d_3/2_ was assigned to four peaks at 410.6, 412.2, 413.6, and 415.0 eV corresponding to Cd^2+^(O), Cd‐Te, Cd‐I, and Cd^2+^(complex), respectively. The I 3d_5/2_ peaks can be fitted into three distinct peaks at 617.7, 619.0, and 620.1 eV, corresponding to the bands of I‐FA, I‐Pb, and I‐Cd, respectively (Figure 2h; Figure S6) [30]. In addition to the chemical interaction between CdTe and perovskite, the construction of p‐n junctions was demonstrated by ultraviolet photoelectron spectroscopy (UPS) and Mott‐Schottky characterizations. As shown in Figure 2i, the electronic structure analysis of the laser‐generated CdTe QDs clearly indicates their p‐type semiconductor characteristics. This is confirmed by an optical bandgap of 2.44 eV (see inset in Figure 2i), which is close to PL peak maximum located at 509 nm (Figure S7), a Fermi energy level (E_F_) of −4.33 eV, a valence band energy level (VB) of −5.25 eV, and a conduction band energy level (CB) of −2.81 eV. When these QDs are embedded in an n‐type perovskite matrix, they are inclined to form p‐n junctions by establishing a consistent E_F_, evidenced by the inverted “V‐shape” plot in the Mott‐Schottky measurement (Figure 2j) [31].

### 2.2 Effect of QDs/Perovskite Microscopic Heterointerfaces on Carrier Dynamics

**FIGURE 3:**
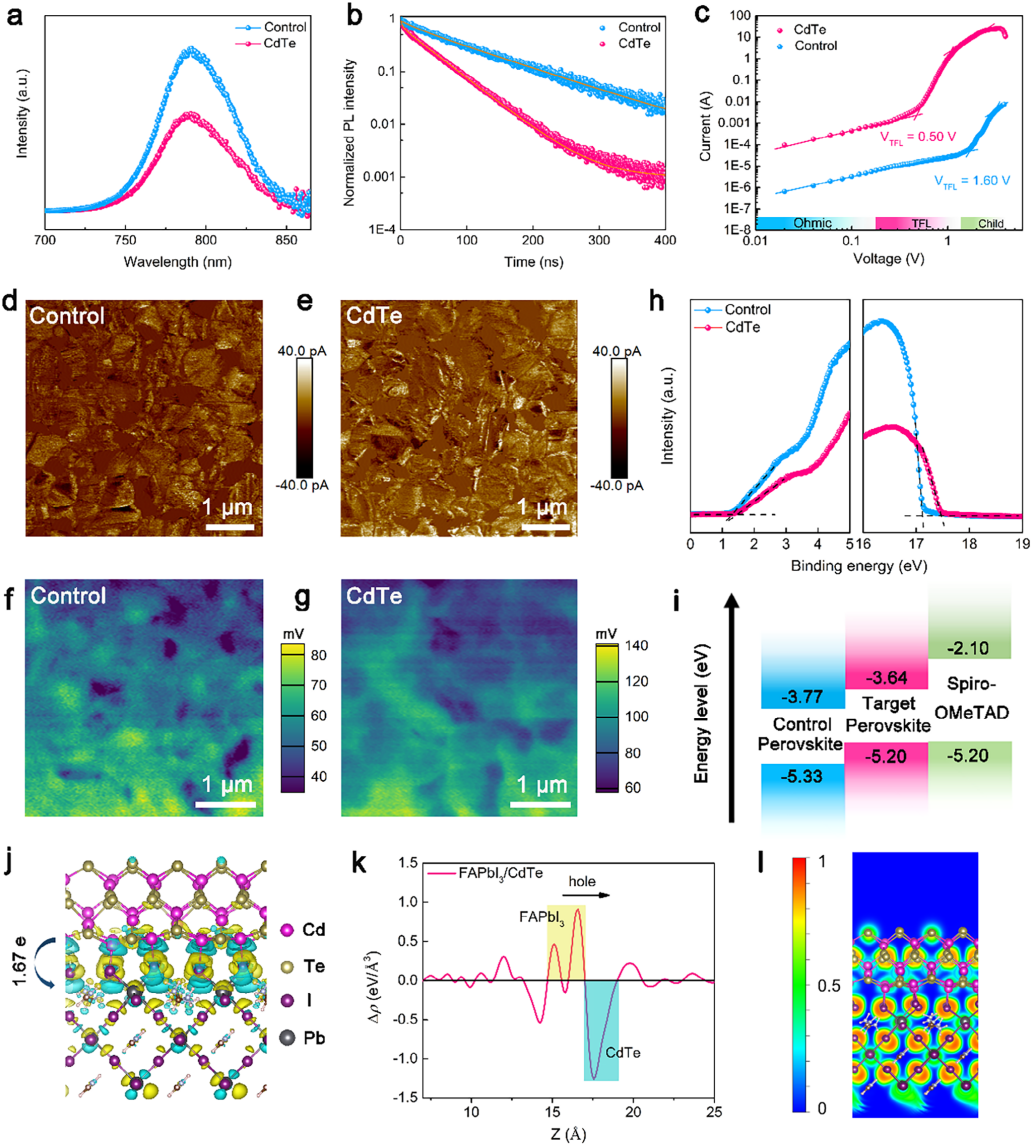
a) Steady‐state and b) time‐resolved PL spectra of different perovskite films. c) SCLC for the hole‐only device. d,e) c‐AFM images of control and CdTe films. f,g) SKPM images of control and CdTe films. h) UPS spectra of different perovskite films. i) Energy level diagram for each component of devices. The energy level of Spiro‐OMeTAD refers to the literature. [36] j) Side view of Δ*ρ* at heterojunction of FAPbI_3_ and CdTe with isovalue of 0.001 eV/Å^3^. Yellow and cyan clouds successively reveal electron accumulation and depletion. k) the Dependence of the Δ*ρ* on the z‐coordinate. l) Slices of ELF at heterojunction of FAPbI_3_ and CdTe.

Steady‐state photoluminescence (PL) and time‐resolved photoluminescence (TRPL) spectra were utilized to examine the carrier dynamics of the perovskite film with and without laser embedded CdTe QDs. Figure 3a shows a significant PL quenching for the CdTe film due to better charge transfer and extraction at the interface of perovskite and CdTe [32]. The carrier lifetimes of different perovskite films, as determined from the TRPL spectra, exhibit an obvious reduction from 98.77 ns for the control film to 43.92 ns for the CdTe film, agreeing well with the PL result (Figure 3b; Table S1). In order to quantitatively evaluate the trap density (*N*_t_) and carrier mobility of various films, the space‐charge‐limited‐current (SCLC) was adopted to record dark *I‐V* curves. Figure 3c presents the trap‐filled limit voltage (*V*_TFL_) of hole‐only devices utilized to determine the *N*_t_, which were calculated to be 4.71 × 10^16^ and 1.47 × 10^16^ cm^−3^ for the control and CdTe films, respectively. This can be attributed to the interaction between CdTe and perovskite that suppresses unwanted defects including iodine vacancies and under‐coordinated Pb^2+^ by facilitating the ionic binding of Cd^2+^ with iodine and coordinating undercoordinated Pb^2+^ defects through Cd^2+^(O) [30]. While noting that there was a notable increase in the hole mobilities extracted from SCLC regions, rising from 0.46 cm^2^ V^−1^ s^−1^ for the control film to 0.87 cm^2^ V^−1^ s^−1^ for the CdTe film (Table S2). Additionally, conductive atomic force microscopy (c‐AFM) and scanning Kelvin probe microscopy (SKPM) were employed to investigate the effect of laser embedding of CdTe QDs on the conductive properties and surface potential (SP) of the perovskite films, which are related to the conductivity and the E_f_ [33, 34]. As illustrated in Figure 3d,e, the CdTe film shows higher conductivity over that of the control film due to significant increase in current across the entire surface [33]. This enhancement can be primarily attributed to the presence of substantial p‐n junctions, which creates an additional pathway at device heterointerface that promotes carrier separation and the oriented transport of photogenerated holes along the CdTe QDs, ultimately facilitating their rapid collection by the hole transport layer [6, 7, 26]. Meanwhile, SKPM images demonstrate a higher average SP of approximately 100 mV for the CdTe film than that of the control film (60 mV), which results in up‐shift of the E_F_ (Figure 3f,g) [35]. This is further corroborated by UPS, which reveals an upward shift in the E_F_, VB, and CB of the perovskite. The energy levels shift from −4.09, −5.33, and −3.77 eV for the control film to −3.76, −5.20, and −3.64 eV for the CdTe films, respectively (Figure 3h). This clear variation in the band structure suggests that the CdTe embedded perovskite film achieves improved energy level alignment with the Spiro‐OMeTAD layer, thereby promoting more efficient hole transport at the upper interface, as shown in Figure 3i.

Furthermore, density functional theory (DFT) calculations were performed to investigate the interfacial interaction between FAPbI_3_ and CdTe. A heterojunction model was constructed using the FAPbI_3_ (022) and CdTe (200) crystallographic planes, as illustrated in Figure S8. The thermodynamic stability of the interface was evaluated by calculating the formation energy, which was determined to be −0.024 eV/Å^2^, indicating strong interactions that favor stable interface formation. The charge density difference (Δ*ρ*) was calculated to visualize charge redistribution at the heterointerface with charge accumulation and depletion (Figure 3j). Figure 3k displays the planar‐averaged Δ*ρ* profile, revealing Δ*ρ* < 0 in the CdTe region adjacent to FAPbI_3_ and Δ*ρ* > 0 at the FAPbI_3_ surface, which is favorable for hole transfer from FAPbI_3_ to CdTe. Bader charge analysis quantified the number of transferred electrons as 1.67 e. The electron localization function (ELF) was further examined to characterize the electron delocalization at the heterojunction. Figure 3l shows significantly localized electron states (red regions) near I atoms of FAPbI_3_, together with high electron delocalization between I and Cd atoms, confirming the ionic character of the Cd─I bonds. This agrees well with the result from Figure 2h, which contributes to robust heterostructure formation and more efficient charge transfer across the heterointerface.

### 2.3 Photovoltaic Performance of Planar PSCs Embedded with CdTe QDs

**FIGURE 4:**
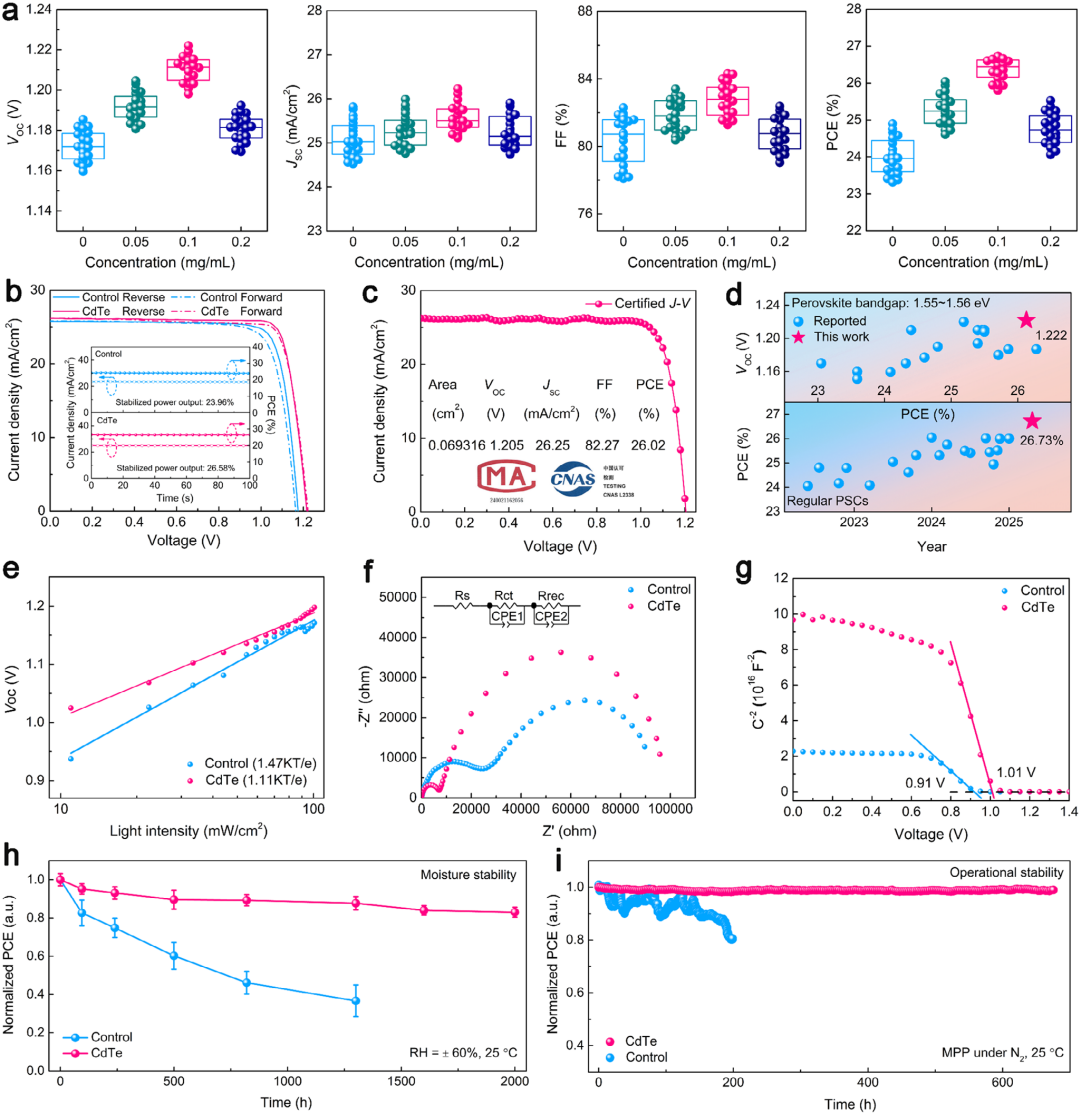
a) The distribution of photovoltaic parameters for 20 individual devices with different concentrations of CdTe QDs. b) *J‐V* curves and stabilized power output at MPP of different champion devices under reverse and forward scanning. c) *J‐V* curves of the best‐performing target device submitted to a CNAS‐accredited certification center. d) Reported values of the PCE from regular PSCs and the *V*_OC_ from PSCs with the bandgap of 1.55–1.56 eV. e) Dependence of the *V*_OC_ on the irradiation intensity, f) Nyquist, and g) Mott–Schottky plots of different devices. h) The humidity stability and i) operational stability of different devices. The error bars represent the standard deviation for 10 devices.

To investigate the impact of embedding CdTe QDs on photovoltaic performance, regular planar PSCs with the configuration of FTO substrate/TiO_2_/perovskite/Spiro‐OMeTAD/Au were fabricated using various QD concentrations. As shown in Figure 4a, statistical distributions of photovoltaic parameters from 20 individual devices indicates that 0.1 mg/mL is the optimal concentration for embedding CdTe QDs. The corresponding parameters were recorded under standard 1 sun illumination (100 mW/cm^2^, AM 1.5G) and are summarized in Table S3. Specifically, the average PCE sharply increases from 24.01% to 26.37%, primarily driven by significant enhancements in *V*_OC_ from 1.172 to 1.210 V and short‐circuit current density (*J*_SC_) from 25.08 mA/cm^2^ to 25.57 mA/cm^2^, as well as fill factor (FF) from 80.38% to 82.76%. Notably, the target device achieved a champion efficiency of 26.73% (calculated by a *V*_OC_ of 1.217 V, a *J*_SC_ of 26.19 mA/cm^2^, and a FF of 83.87%) under reverse scan of the current density‐voltage measurement with negligible hysteresis (1.35%) and a stabilized power output of 26.58%, significantly outperforming the control device's 24.90% efficiency and 23.96% stabilized output (Figure 4b and Table 1). As shown in Figure 4c, the target device, submitted to a CNAS‐accredited certification center, achieved a certified PCE of 26.02%, with a *V*_OC_ of 1.205 V, a *J*_SC_ of 26.25 mA/cm^2^, and a FF of 82.27% (Figure S9). The EQE spectra of different champion devices deliver integrated current densities of 24.60 and 25.11 mA/cm^2^, respectively, agreeing well the *J*_SC_ from *J‐V* results (Figure S10). The enhancement of EQE response in the entire wavelength range is mainly attributed to the incorporation of CdTe QDs into the perovskite, which induces vertically oriented grain growth throughout entire perovskite film (Figure 2b), resulting in the fabrication of high‐crystallinity film [35, 36]. It is worth noting that champion efficiency of 26.73% achieved in present work ranks among the highest reported for regular PSCs, accompanied by a record‐high *V*_OC_ with a remarkably low voltage deficit of 0.338 V at a bandgap of 1.56 eV (Figure 4d; Figure S11, Tables S4 and S5). Moreover, in order to confirm the scalability of the LSPC process on fabricating PSC with a larger active area, the 1‐cm^2^ device yielded a remarkable efficiency of 25.45%, with a *V*_OC_ of 1.184 V, a *J*_SC_ of 25.94 mA/cm^2^, and a FF of 82.87% (Figure S12).

**TABLE 1:** The Photovoltaic parameters of different champion devices with and without CdTe QDs embedding under forward and reverse scanning.

Scanning direction	*V*_OC_ (V)	*J*_SC_ (mA/cm^2^)	FF (%)	PCE (%)	Hysteresis index (%)
Control Forward	1.164	25.82	79.34	23.85	4.22
Control Reverse	1.175	25.77	82.24	24.90	
CdTe Forward	1.222	26.17	82.47	26.37	1.35
CdTe Reverse	1.217	26.19	83.87	26.73	

The hysteresis index is calculated according the equation: Hysteresis index = (PCE_Reverse_‐PCE_Forward_)/PCE_Reverse_.

To further gain a deeper understanding of the mechanisms behind the improved photovoltaic performance, the related carrier recombination dynamics within the device were systematically investigated. Figure 4e shows that *V*_OC_ plots versus light intensity reveal a significant reduction in fitted slopes, from 1.47 kT/e for the control device to 1.11 kT/e for the target device. This result indicates effective suppression of trap‐assisted recombination within the target device, which can be ascribed to the formation of p‐n junctions that accelerate charge separation, and reduced defect‐induced carrier trapping upon passivation effect of both iodide vacancy and undercoordinated Pb^2+^. As further confirmed by the electrical impedance spectroscopy (EIS), Figure 4f illustrates a decrease in the charge transport resistance (*R*_ct_) from 24358 Ω in the pristine device to 6858 Ω in the target device, alongside an increase in the recombination resistance (*R*_rec_) from 89068 to 96976 Ω (Table S6). This demonstrates enhanced charge transport and inhibited charge recombination in the perovskite film following laser embedding of CdTe QDs. Such encouraging results suggest a potential increase in *V*_OC_, as evidenced by Mott‐Schottky curves of the control and target devices, which show built‐in potentials of 0.91 and 1.01 V, respectively (Figure 4g). Additionally, the lower interfacial charge density inferred from the slope of curve for target device reveals enhanced hole extraction at the interface of perovskite and Spiro‐OMeTAD, in agreement with the UPS result.

### 2.4 Environmental Stability of PSCs with Embedded CdTe QDs

Further evaluation of the environmental stability of PSCs was carried out to further investigate the effect of laser embedding of CdTe QDs on their long‐term durability under varying testing conditions. The long‐term humidity stability of unencapsulated PSCs was evaluated in a chamber maintained at 60% relative humidity, as illustrated in Figure 4h. The results reveal that the PSCs embedding CdTe QDs show significantly enhanced resistance to moisture compared to the control devices, retaining 83% of their initial PCE after 2000 h, whereas the PCE of control devices dropped to below 40% after just 1300 h. This is primarily due to synergistic effects where the chemical passivation via Cd‐I/Cd^2+^(O) bonds stabilizes the film at the atomic level by removing decomposition initiation sites, while CdTe‐induced heteroepitaxial growth enables the construction of robust interfacial microstructures and further high‐quality films slow down moisture ingress. Likewise, the target devices exhibit excellent thermal stability, reflecting from lower PCE degradation (less than 14% of initial PCE) under persistent heat treatment at 85°C than that of the control devices (over 46%), as shown in Figure S13. Figure 4i further illustrates the operational stability of various champion devices while tracking the MPP under 1 sun illumination in an N_2_ atmosphere without a UV filter. After suffering from approximately 200 h of illumination, the control device degrades to 83% of its initial PCE. In contrast, the target device remarkably retains over 98% of its initial PCE even after almost 700 h. This impressive stability is attributed to the effective passivation of undercoordinated Pb^2+^ ions by Cd^2+^(O), as well as the restrained migration of iodide ions facilitated by Cd‐I ionic bindings.

## 3 Discussion

Driven by significant challenges of synthesizing well‐defined QDs in desired antisolvents and the limitations posed by surface‐grafted ligands in wet‐chemical methods, which often hinder charge transfer, we demonstrate a LSPC technique that produces ligand‐free QDs directly in antisolvents to pursue high‐performance PSCs [37]. Subsequent embedding of multifunctional CdTe QDs in perovskite films plays several significant roles in enhancing the efficiency and stability of PSCs. First, the CdTe QDs exhibit their p‐type semiconductor characteristics, enabling the formation of p‐n junctions through establishing a consistent E_F_ and gradient band alignment due to their tailored size when embedded in the n‐type perovskite matrix. This results in not only the creation of substantial and unidirectional BEFs that promote the directional transport of photogenerated holes at device heterointerfaces, but the increased depletion region that minimizes recombination loss [38]. Second, such QDs could serve as efficient passivation agents, effectively mitigating defects by promoting the ionic binding of Cd^2+^ with iodine that suppresses iodine ion migration and passivating undercoordinated Pb^2+^ through Cd^2+^(O) at both surface and GBs, which principally account for shallow hole potential wells and improved environmental stability. Third, the exceptionally low lattice mismatch of 2.86% between CdTe (a = 6.48 Å) and perovskite (a = 6.30 Å) facilitates coherent heterointerface formation. Moreover, the embedded CdTe QDs trigger uniform crystallization toward favorable grain orientation (Figures S4 and S14). These collective effects contribute to construction of high‐quality perovskite films with robust microstructures. The CdTe QDs also improve the energy level alignment between the perovskite and Spiro‐OMeTAD, further enhancing hole extraction at the top interface. Leveraging these synergistic merits, we thus achieved a target PSC with a top record PCE of 26.73% (certified 26.02%) among regular PSCs (Table S4), along with stable shelf storage over 2000 h (RH of 60%) and remarkable light stability at the MPP that exhibits nearly no efficiency degradation over 600 h. While noting that a record‐high *V*_OC_ of 1.222 V corresponds to a remarkably low voltage deficit of 0.338 V at a bandgap of 1.56 eV (Table S5). The significant enhancement in all photovoltaic parameters indicates that the increases in *V*_OC_, *J*_SC_, and FF are primarily due to improved energy level alignment at the top interface, reduced nonradiative recombination, efficient charge extraction, and decreased charge transport resistance within the perovskite film, respectively. This study highlights significant potential of multifunctional p‐type semiconducting QDs on constructing the microscopic heterointerfaces, accessing state‐of‐the‐art PSCs with triple managements of carrier dynamics, crystallization kinetics, and defect manipulation.

## 4 Conclusion

In summary, this work demonstrates an innovative strategy to construct coherent microscopic heterointerface through embedding well‐defined p‐type CdTe QDs into an n‐type perovskite matrix for boosted carrier management, photovoltaic efficiency, and long‐term durability of PSCs. The surface embedding of CdTe enables the formation of p‐n heterojunctions with homogeneous distribution that establishes unidirectionally aligned BEFs to promote directional charge transport while suppressing recombination loss through expanded depletion regions. Near‐perfect lattice matching enabling dislocation‐less interfaces between CdTe and perovskite, along with effective defect passivation of iodine vacancies and undercoordinated Pb^2+^ at both surface and GBs, yield high‐crystallinity films and robust PSCs. The resultant PSC deliver a record‐high efficiency of 26.73% (certified 26.02%) and an outstanding *V*_OC_ of 1.222 V at a 1.56 eV bandgap, along with remarkable operational stability that maintains over 98% of their initial efficiency after nearly 700 h under maximum power point tracking and stable shelf storage over 2000 h (RH of 60%). The present work pioneers a new paradigm in constructing microscopic heterointerface that can simultaneously manipulate carrier management and operational stability in advanced optoelectronics.

## Author Contributions

Feng Yan and Hongqiang Wang have proposed the concept and directed the research. Pengfei Guo has directed DFT and preparation of devices. Yadong Xu has proposed the suggestions on the research. Wenhao Zhao performed the preparation of materials and devices and completed the manuscript. Wenhao Zhao, Deyou Lin, Riming Sun, and Zhiyu Fang performed the characterization of materials and devices. All the authors have participated in the discussion of the results.

## Conflicts of Interest

The authors declare no conflicts of interest.

## Supporting information




**Supporting file 1**: adma72124‐sup‐0001‐SuppMat.docx.

**Supporting file 2**: adma72124‐sup‐0002‐DataFile.zip.

## Data Availability

Research data are not shared.
