# Supplementary material for: CdTe Quantum Dots Encapsulated on Perovskite Grains Enable Highly Efficient and Stable Perovskite Solar Cells
Source: Adv Mater. 2026 Jan 10;38(12):e21104. doi: 10.1002/adma.202521104 (PMC12933008; doi:10.1002/adma.202521104)
Supplement: Supplementary file 1 — Supporting file 1: adma72124‐sup‐0001‐SuppMat.docx. [file ADMA-38-e21104-s001.docx]

**CdTe Quantum Dots Encapsulated on Perovskite Grains Enable Highly Efficient and Stable Perovskite Solar Cells**

*Wenhao Zhao, Deyou Lin, Riming Sun, Zhiyu Fang, Pengfei Guo^*^,* *Yadong Xu, Hongqiang Wang^*^* *and Feng Yan^*^*

Dr. W. Zhao, D. Lin, R. Sun, Z. Fang, Dr. P. Guo, Dr. Y. Xu, Prof. H. Wang

State Key Laboratory of Solidification Processing, Center for Nano Energy Materials, School of Materials Science and Engineering, Northwestern Polytechnical University and Shaanxi Joint Laboratory of Graphene (NPU), Shaanxi Laboratory for Advanced Materials, Xi’an, 710072, P. R. China

Dr. W. Zhao, Prof. F. Yan

Department of Applied Physics, Research Centre for Organic Electronics, The Hong Kong Polytechnic University, Hung Hom, Kowloon, Hong Kong P. R. China

Dr. P. Guo

Research & Development Institute of Northwestern Polytechnical University in Shenzhen, Shenzhen, 518063, P. R. China

E-mail: [guopengfei@nwpu.edu.cn;](mailto:guopengfei@nwpu.edu.cn;) [hongqiang.wang@nwpu.edu.cn](mailto:hongqiang.wang@nwpu.edu.cn); apafyan@polyu.edu.hk

**Experimental Section**

**Materials**

All chemicals and materials were used as received unless otherwise specified. Fluorine-doped tin oxide (FTO) coated glass substrates (sheet resistance ~20 Ω/sq) with partial etching were purchased from OPV Tech. Bulk CdTe precursor materials and organic solvents including dimethylformamide (DMF), dimethyl sulfoxide (DMSO), chlorobenzene (CB), ethyl acetate (EA) and acetonitrile (ACN) were obtained from Sigma-Aldrich. All other materials were purchased from Xi'an Polymer Light Technology Corp.

**Preparation of ligand-free CdTe quantum dots (QDs) in desired solvent**

The CdTe nanocolloids were prepared through non-focusing nanosecond pulsed laser irradiation (Quantel, repetition rate: 10 Hz, pulse width: 8 ns, beam diameter: 8 mm, fluence range: 300~600 mJ/pulse cm^2^) with a wavelength of 1064 nm and fixed irradiation time of 3 min. Briefly, bulk CdTe were immersed in 10 mL EA within a temperature-controlled reaction chamber maintained at -20 °C. The bulk CdTe was irradiated along with continuous ultrasonic treatment to ensure the homogeneity of well-dispersed CdTe QDs. The concentration of CdTe colloids ranges from 0.05 to 0.3 mg/mL.

**Device Fabrication**

FTO-coated glass substrates were sequentially cleaned with detergent, deionized water, acetone, isopropanol, and ethanol under ultrasonic treatment for 15 min each. The substrates were then dried under N₂ flow and treated with ozone plasma for 10 minutes to enhance surface wettability. A 50 nm TiO₂ compact layer was deposited via chemical bath deposition using an aqueous TiCl₄ solution (2.25:100 v/v TiCl₄:H₂O) at 70 °C for 1 h, followed by annealing at 150 °C for 1 h. Then Cs_0.05_FA_0.85_MA_0.10_PbI_2.91_Br_0.09_ precursor solution (1.55 M) was prepared in a DMF:DMSO (4:1 v/v) mixture at 55°C for 2 hours. The solution was spin-coated in a N₂ glovebox using a one-step program (4000 rpm for 30 s) with the EA anti-solvent dripping 10s prior the end of the program. In particular, various concentrations of CdTe (0.1, 0.2, and 0.3 mg/mL) were dispersed in the EA, subsequent annealing of the films were conducted at 150 °C for 1 h. Then PEAI (3 mg/mL in isopropanol) was spin-coated at 5000 rpm for 30 s on the perovskite layer. Spiro-OMeTAD solution was prepared by mixing 72 mg Spiro-OMeTAD, 29 μL tBP, and 18 μL Li-TFSI stock solution (520 mg/mL in ACN) in 1 mL CB, then spin-coated at 6000 rpm for 30 s. 100 nm Au electrodes were thermally evaporated through a shadow mask, defining an active area of 0.09 cm². A 0.0691 cm² non-reflective mask and an anti-reflective film were used during current density-voltage (*J-V*) test.

**Characterization**

High-resolution transmission electron microscopy (HRTEM) with EDS were conducted employing an FEI Tecnai F30 transmission electron microscope operated at 300 kV, equipped with an Oxford Instruments EDS detector and a high angle annular dark field (HAADF) STEM detector. Field emission scanning electron microscopy (SEM, FEI Nova) was employed for morphological analysis. Atomic force microscopy (AFM, Bruker Dimension Icon) was employed for the analysis of surface topography, along with scanning Kelvin probe microscopy (SKPM) and conductive-AFM (c-AFM) for measurement of surface potential and conductive properties. X-ray diffraction (XRD, PANalytical X'pert PRO) was conducted using Cu Kα as the X-ray source (λ = 0.15406 nm). X-ray photoelectron spectroscopy (XPS, Kratos Axis Supra) was characterized for elemental composition and chemical state determination. Ultraviolet photoelectron spectroscopy (UPS) was characterized using HeI emission line (21.22 eV) with VG Scienta R4000 analyzer. Steady-state photoluminescence (PL) and time-resolved PL (TRPL) spectra were obtained using 470 nm pulsed laser excitation (Horiba FluorologFL-3). In situ PL monitoring was performed during film growth using 532 nm laser diode and visible range spectrometer. The *J-V* and steady-state output were measured under AM 1.5G illumination (100 mW/cm²) using a xenon lamp solar simulator (Oriel 67005) and Keithley 2400 source meter, calibrated with a monocrystalline silicon reference cell (Hamamatsu S1133). Dark current-voltage curves were recorded using a Keithley 2400 source for space-charge-limited current (SCLC) analysis. Electrochemical impedance spectroscopy (EIS, CHI660E) was characterized from 1 MHz to 0.1 Hz at 0.8 V bias in the dark. Mott-Schottky analysis was carried out by recording capacitance-voltage curve at 1 kHz frequency with an applied bias voltage from 0 V to 1.4 V (CHI660E). External quantum efficiency (EQE) measurements were conducted using an Enlitech QE-R3011 system. During long-term stability measurements, a superoxide radical derived metal-free Spiro-OMeTAD according to our previous report was used as hole transport layer within the perovskite solar cells (PSCs) to enhance resistance upon environmental stimuli.^[1]^

**Statistical Analysis:** All the quantitative experiments were performed at least three replicates for each group to ensure reproducibility. All the quantitative values are presented as means ± standard deviation. The statistical analysis was carried out adopting the *t*-test, and statistical significance was determined using a p-value of less than 0.05. The error bars represent the standard deviation of data points from independent samples.

**Density Functional Theory (DFT) Calculations**

All the calculations are performed in the framework of the density functional theory with the projector augmented plane-wave method, as implemented in the Vienna ab initio simulation package.^[2]^ The generalized gradient approximation proposed by Perdew, Burke, and Ernzerhof is selected for the exchange-correlation potential.^[3]^ The long range van der Waals interaction is described by the DFT-D3 approach.^[4]^ The cut-off energy for plane wave is set to 400 eV. A vacuum layer of 12 Å is added perpendicular to the sheet to avoid artificial interaction between periodic images. During geometry optimization, all atomic coordinates were relaxed with the convergence criteria of 0.03 eV·Å^-1^ for the forces on each atom, except for the atoms in the bottom two layers of the slab. For each self-consistent iteration, the total electronic energy was converged to 10^-5^ eV. The Brillouin zone integration is performed using a 1×1×1 k-mesh. The charge density diﬀerence was calculated as $\boldsymbol{\Delta\rho(r)}=\boldsymbol{\rho}^{CdTe/FAPbI3}-\boldsymbol{\rho}^{CdTe}-\boldsymbol{\rho}^{FAPbI3}$, where $\boldsymbol{\rho}^{CdTe/FAPbI3}$ is the electron density of the interacting system, while $\boldsymbol{\rho}^{CdTe}$ and $\boldsymbol{\rho}^{FAPbI3}$ are the electron densities of the CdTe (200) surface and FAPbI_3_ (022) slab calculated for the isolated systems with the atoms fixed in their positions for molecule/slab composite cell. The electron density diﬀerence isosurfaces were visualized with VESTA.^[5]^ The interface formation energy (*E*_f_) was calculated as:
*E*_f_ = *E*_FAPbI3/CdTe_ - *E*_FAPbI3_ - *E*_CdTe_, where *E*_FAPbI3/CdTe_, *E*_FAPbI3_ and *E*_CdTe_ are the energy of calculated FAPbI_3_/CdTe, FAPbI_3_ (022) and CdTe (200).


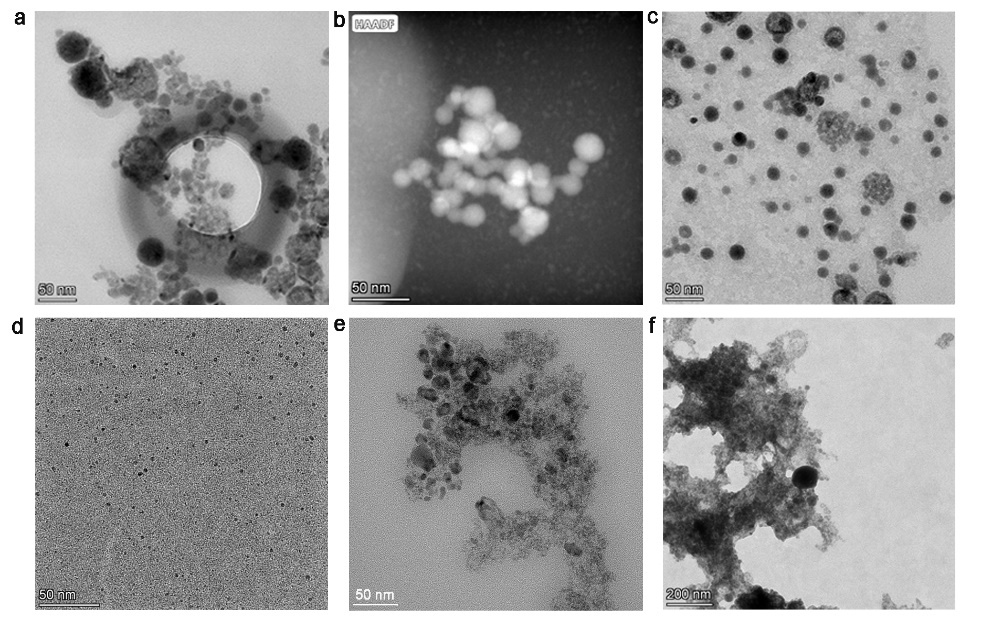


**Figure S1.** Producing CdTe particles by different laser fluence of (a) 300, (b) 400, and (c) 500 mJ pulse^-1^ cm^-2^ enables gradually decrease in particle size. TEM images of different concentration of CdTe particles with (d) 0.05, (e) 0.2, and (f) 0.3 mg/ml.

The well-dispersed ligand-free CdTe QDs with sizes below 10 nm in this work were synthesized by optimizing the laser fluence (300~600 mJ/pulse·cm²) and concentration (0.05~0.3 mg/ml) while maintaining fixed irradiation time and cooling temperature. For laser fluence optimization, raising the fluence from 300 to 500 mJ/pulse·cm² led to a progressive reduction in the overall size of the spherical particles, attributed to laser-induced fragmentation in liquid (Figure S1a-c). A tailored laser fluence range of 300~600 mJ/pulse·cm² was employed to produce CdTe nanomaterials of varying sizes, with the optimal fluence of 600 mJ/pulse·cm² yielding well-dispersed CdTe QDs (Figure 1b).

The concentration of target materials also plays a crucial role in forming monodisperse QDs. When the concentration of raw CdTe particles increases from 0.05 to 0.2 mg/ml and further to 0.3 mg/ml, the laser-generated CdTe nanoparticles transition from a well-dispersed to an agglomerated state, as evidenced in Figure S1d-f. The embedding of such agglomerated CdTe nanoparticles would keep their agglomerated state in the perovskite film and result in the deterioration of photovoltaic property of PSCs, as shown in Figure 4a and Table S3, Supporting Information. Therefore, the optimal CdTe concentration for embedding was determined to be 0.1 mg/ml.


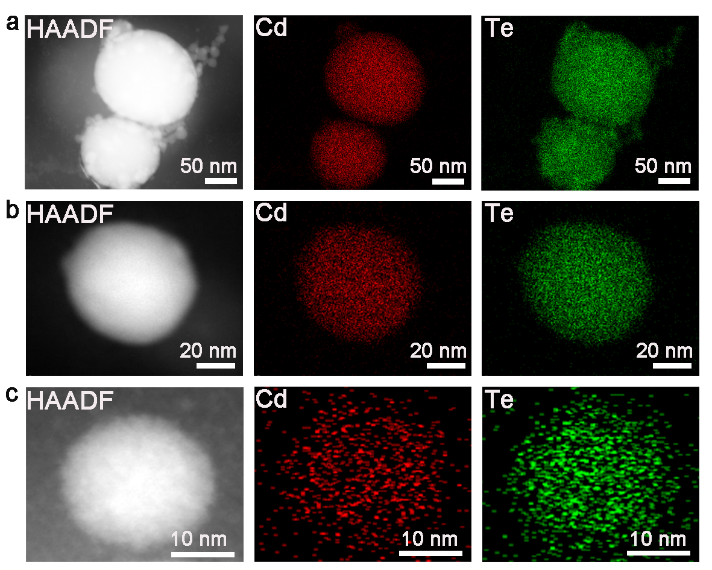


**Figure S2.** HAADF and corresponding TEM-EDS images of different size of CdTe nanoparticles.


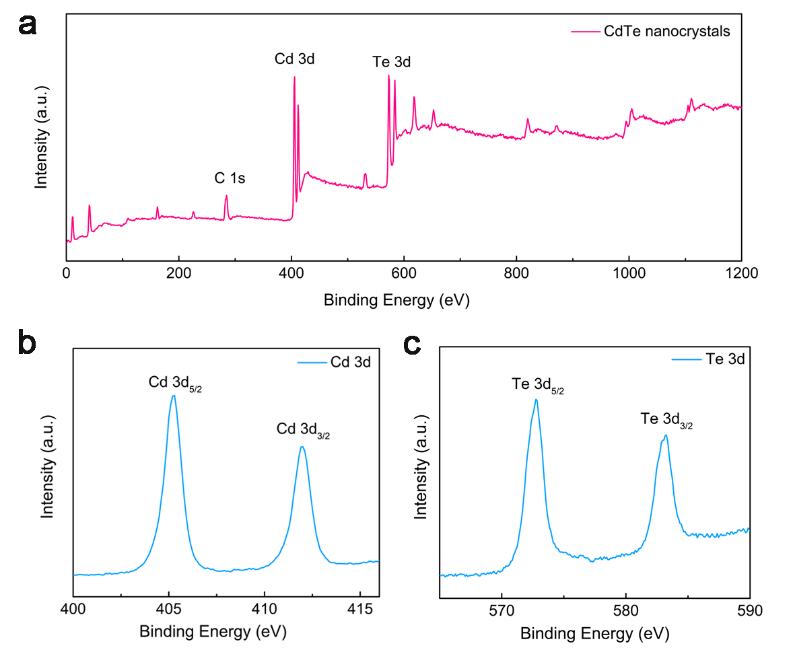


**Figure S3.** XPS spectrum (a) and high-resolution XPS spectra (b) of CdTe QDs.

The presence of characteristic XPS peaks for Cd 3d_3/2_ at 412.0 eV, Cd 3d_5/2_ at 405.2 eV, Te 3d_5/2_ at 572.8 eV, and Te 3d_3/2_ at 583.2 eV confirms the successful formation of CdTe QDs. Notably, no significant shifts in these peaks were observed after laser irradiation, indicating that the surface composition and chemical states of CdTe remained unchanged.^[6]^





**Figure S4.** XRD patterns of different perovskite films.





**Figure S5.** ToF-SIMS depth profiles of the perovskite film with CdTe embedding.


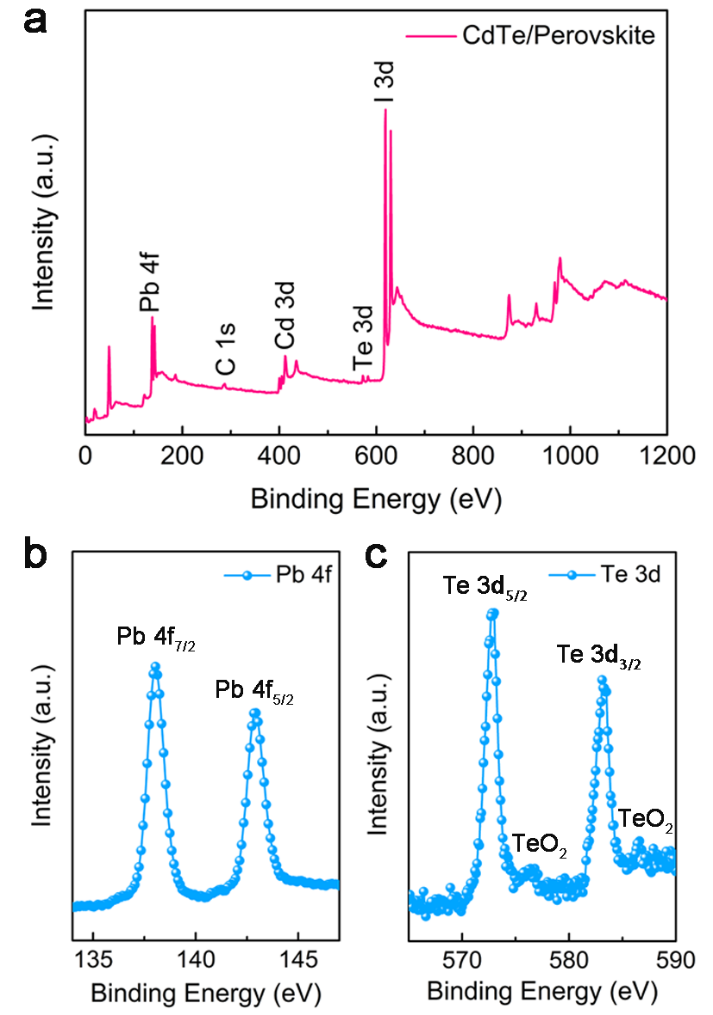


**Figure S6.** (a) XPS spectrum, high-resolution XPS spectra of (b) Pb 4f and (c) Te 3d of perovskite film with CdTe QDs embedding. The results show that XPS peaks of Pb 4f are observed at 138.1 and 142.9 eV, respectively. Meanwhile, XPS peaks of Te 3d are observed at 572.8, 583.2 eV that correspond to Cd-Te, and XPS peaks of 576.1 and 586.5 eV is assigned to TeO_2_.

**Table S1.** Summaries of parameters extracted from the fitted plots of the corresponding TRPL spectra following bi-exponential rate law: f(t) = *A*_1_exp(-t/*τ*_1_ )+*A*_2_exp(-t/*τ*_2_ )+y_0_, where *A*_1_ and *A*_2_ represent the relative amplitudes, *τ*_1_ represents trap-assisted recombination, and *τ*_2_ represents free carrier recombination. The *τ*_ave_ is calculated using *τ*_ave_ = $\frac{\sum A_{i}\tau i^{2}}{\sum A_{i}\tau i}$ formula.^[7]^

| Samples | *A*_1_ | *τ*_1_ (ns) | *A*_2_ | *τ*_2_ (ns) | *τ*_ave_ (ns) |
| --- | --- | --- | --- | --- | --- |
| Control | 0.25 | 24.55 | 0.75 | 104.58 | 98.77 |
| CdTe | 0.20 | 3.96 | 0.80 | 44.80 | 43.92 |

**Table S2** The values of *V*_TFL_ and calculated trap densities (*N*_t_) for control and target devices.

| Samples | *V*_TFL_ (V) | *N*_t_ (cm^-3^) | µ (cm² V^-^¹ s^-1^) |
| --- | --- | --- | --- |
| Control | 1.60 | 4.71$\times$10^16^ | 0.46 |
| CdTe | 0.50 | 1.47$\times$10^16^ | 0.87 |

The *N*_t_ of different perovskite films for electron-only devices were evaluated using $N_{t}=\frac{2\varepsilon_{0}\varepsilon V_{\mathrm{TFL}}}{qL^{2}}$ formula, where$\varepsilon_{0}$is the vacuum permittivity, $\varepsilon$ is the relative dielectric constant, $V_{\mathrm{TFL}}$ is the onset voltage of the trap-filled limit region, *q* is the elemental charge, and *L* is the thickness of the perovskite layer.^[8]^ We fabricated a hole-only device with a structure of FTO/PEDOT/perovskite/Spiro-OMeTAD/Au to obtain hole mobility (µ) adopting µ = 8*J*_D_*L*^3^/9*ƐƐ*_0_*V*^2^ formula,^[8]^ where *J*_D_, *V*, and *L* are the dark current density, applied voltage, and the thickness of the perovskite film, respectively.





**Figure S7.** The photoluminescence (PL) spectrum of the pure CdTe colloidal solution.


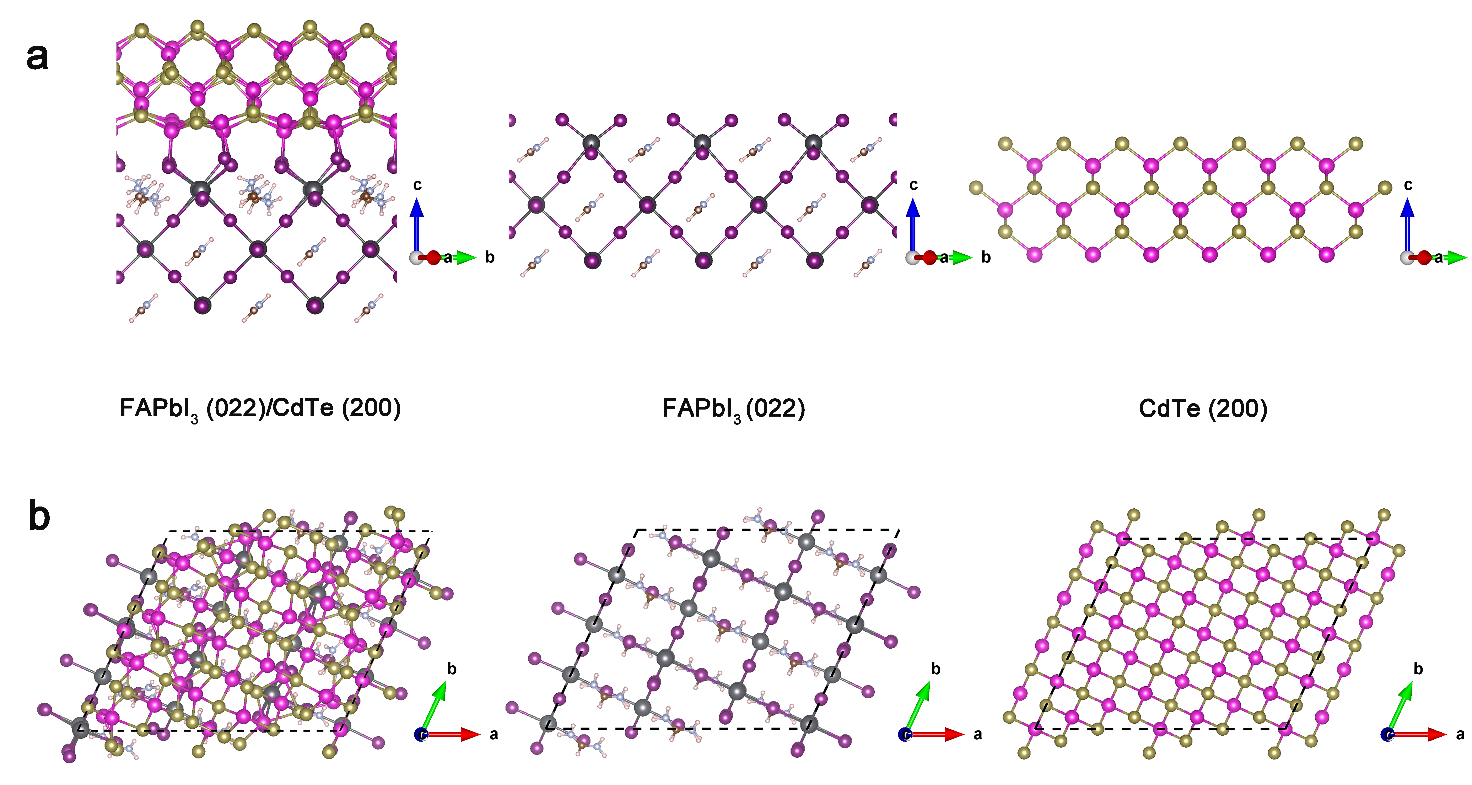


**Figure S8.** Schematic illustration of the interaction between FAPbI_3_ and CdTe as well as FAPbI_3_ and CdTe with a) side view and b) top view.


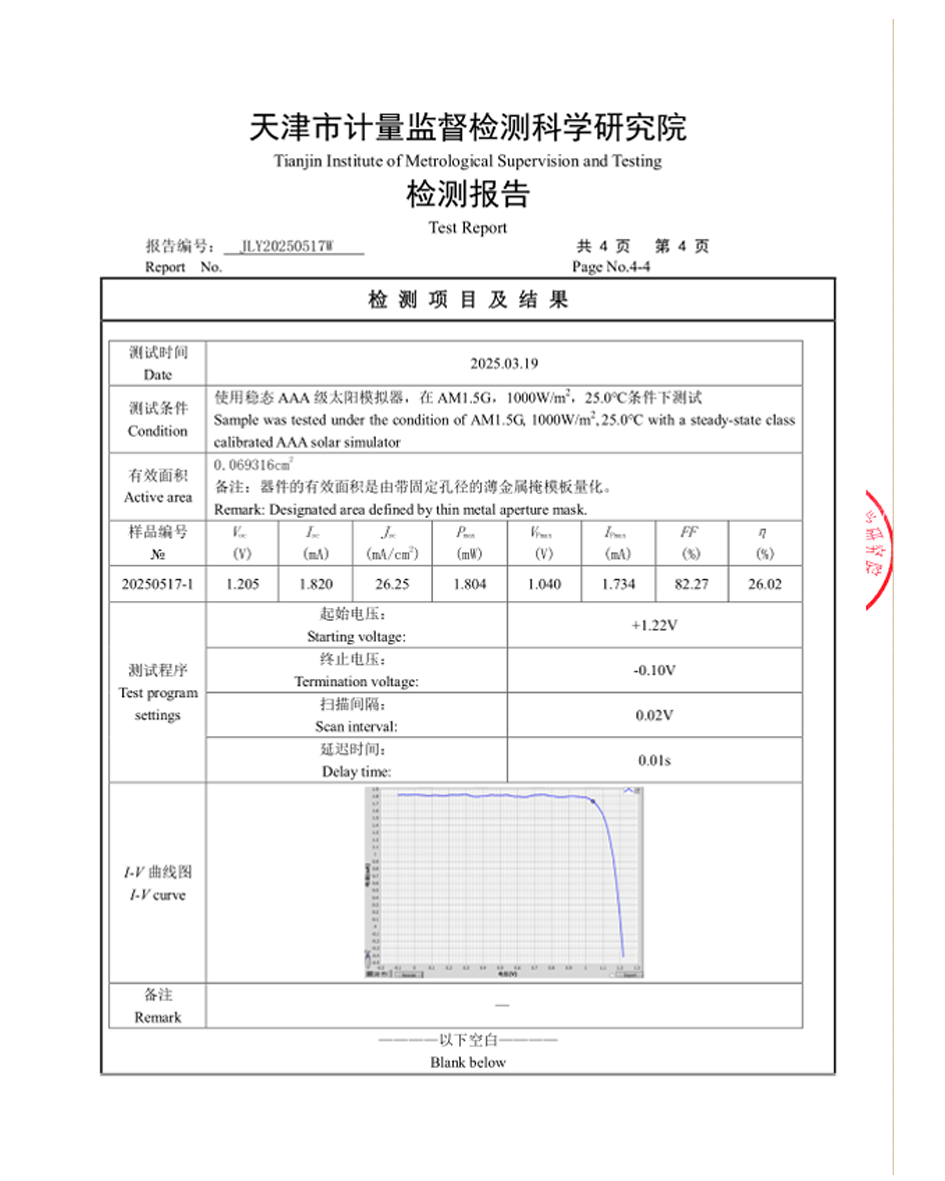


**Figure S9.** Certified efficiency of champion device submitted to a CNAS-accredited certification center.





**Figure S10.** The EQE spectra and the integrated current density of different champion devices.

**Table S3.** Photovoltaic parameters of champion PSCs with different concentration of CdTe QDs.

| Active layers | Concentration of CdTe (mg/mL) | Average  *V*oc  (V) | Average  *J*sc  (mA/cm^2^) | Average  FF  (%) | Average  PCE  (%) |
| --- | --- | --- | --- | --- | --- |
| Control | 0 | 1.172 ± 0.007 | 25.08 ± 0.36 | 80.38 ± 1.28 | 24.01 ± 0.42 |
| 1-CdTe | 0.05 | 1.192 ± 0.006 | 25.27 ± 0.28 | 81.84 ± 0.87 | 25.26 ± 0.36 |
| 2-CdTe | 0.1 | 1.210 ± 0.005 | 25.57 ± 0.20 | 82.76 ± 0.83 | 26.37 ± 0.30 |
| 3-CdTe | 0.2 | 1.181 ± 0.005 | 25.24 ± 0.35 | 80.72 ± 0.91 | 24.76 ± 0.37 |





**Figure S11.** The Tauc plot of perovskite film for extraction of the bandgap.

**Table S4** Reported values for reported PCE from regular planar PSCs.

| **Perovskite** | **Additive** | **Champion**  **PCE (%)** | **Ref.** |
| --- | --- | --- | --- |
| FAPbI_3_ | MXenes | 24.17 | [9] |
| FAPbI_3_ | KHSO | 24.09 | [10] |
| FAPbI_3_ | fluoro-N,N,N″,N″-tetramethylformamidinium hexafluorophosphate | 24.62 | [11] |
| FA_0.85_MA_0.1_Cs_0.05_PbI_3_ | iodobenzene diacetate | 25.76 | [12] |
| FAPbI_3_ | PAA | 24.06 | [13] |
| FA_0.92_MA_0.08_PbI_3_ | LD-TiO_2_ | 24.81 | [14] |
| FAPbI_3_ | Ti_0.936_O_2_ | 25.50 | [15] |
| CsFAPb(IBr)_3_ | C-PCBA | 24.80 | [16] |
| FAPbI_3_ | TG | 26.02 | [17] |
| FACsPbI_3_ | NMS | 24.95 | [18] |
| Cs_0.05_FA_0.85_MA_0.10_PbI_2.91_Br_0.09_ | Eu(TFSI)_2_ | 25.45 | [1] |
| FA_0.85_MA_0.1_Cs_0.05_PbI_3_ | H_2_O | 26.00 | [19] |
| FAPbI_3_ | poly-  hexamethyleneguanidine hydrochloride | 25.42 | [20] |
| FAPbI_3_ | guanabenz acetate salt | 25.32 | [21] |
| FAPbI_3_ | 4-chloro-3-sulfamoylbenzoic acid | 25.32 | [22] |
| FA_0.85_MA_0.1_Cs_0.05_PbI_3_ | tetrahydrothiophene 1-oxide | 26.05 | [23] |
| FA_0.98_MA_0.02_PbI_3_ | 4-tert-butylcalix[8]arene | 26.01 | [24] |
| Cs_0.05_FA_0.85_MA_0.10_PbI_2.91_Br_0.09_ | CdTe | 26.73 | **Our work** |

**Table S5** Reported values of the *V*_OC_ from PSCs with the bandgap of 1.55~1.56 eV.

| **Perovskite** | **Additive** | ***V*_OC_**  **(V)** | **Ref.** |
| --- | --- | --- | --- |
| Cs_0.05_FA_0.85_MA_0.10_PbI_2.9_Br_0.1_ | KHSO | 1.151 | [10] |
| Cs_0.05_MA_0.15_FA_0.80_PbI_3_ | DC-PA | 1.160 | [25] |
| FA_0.9_Cs_0.1_PbI_3_ | BAH | 1.220 | [26] |
| FACsPbI_3_ | AT | 1.180 | [27] |
| Cs_0.05_(FA_0.95_MA_0.05_)_0.95_Pb  (I_0.95_Br_0.05_)_3_ | Al_2_O_3_ | 1.208 | [28] |
| Cs_0.05_(FA_0.98_MA_0.02_)_0.95_  Pb(I_0.98_Br_0.02_)_3_ | β-poly(1,1-difluoroethylene) | 1.177 | [29] |
| Cs_0.05_(MA_0.05_FA_0.95_)_0.95_ Pb(I_0.95_Br_0.05_)_3_ | PEIE | 1.170 | [30] |
| Cs_0.06_MA_0.14_FA_0.80_PbI_3_ | Ammonium ligands | 1.160 | [31] |
| Cs_0.06_MA_0.14_FA_0.80_PbI_3_ | GBAC | 1.190 | [32] |
| Cs_0.05_(FA_0.95_MA_0.05_)_0.95_Pb(I_0.95_Br_0.05_)_3_ | MPA-CPA | 1.210 | [33] |
| CsFAMAPbI_X_Br_3-X_ | DMAcPA | 1.187 | [34] |
| Cs_0.05_(MA_0.05_FA_0.95_)_0.95_ Pb(I_0.95_Br_0.05_)_3_ | CB-NH_2_ | 1.170 | [35] |
| (CsPbI_3_)_0.05_[(FAPbI_3_)_0.97_  (MAPbBr_3_)_0.03_]_0.95_ | poly-BCP | 1.210 | [36] |
| Cs_0.05_FA_0.85_MA_0.1_PbI_3_ | bimolecular SAM | 1.187 | [37] |
| MAFAPbI_X_Br_3-X_ | stage A-ii SnO_2_ | 1.194 | [38] |
| FA_0.9_Cs_0.1_PbI_3_ | TFSAP | 1.210 | [7] |
| Cs_0.05_FA_0.85_MA_0.10_PbI_2.91_Br_0.09_ | Eu(TFSI)_2_ | 1.210 | [1] |
| Cs_0.05_FA_0.85_MA_0.10_PbI_2.91_Br_0.09_ | CdTe | 1.222 | **Our work** |





**Figure S12.** *J-V* curves of the best-performing target device with active area of 1 cm^2^.

**Table S6** Summaries of parameters extracted from the fitted plots of the impedance spectra of devices with and without laser embedding of CdTe QDs by using an equivalent circuit model.

| Samples | *R*s  (Ω) | *R*ct  (Ω) | CPE1-T  (F) | CPE1-P  (F) | *R*rec  (Ω) | CPE2-T  (F) | CPE2-P  (F) |
| --- | --- | --- | --- | --- | --- | --- | --- |
| Control | 28.25 | 24358 | 2.82E-9 | 0.85 | 89068 | 2.32E-6 | 0.73 |
| CdTe | 22.57 | 6858 | 7.78E-9 | 0.95 | 96976 | 1.29E-6 | 0.80 |





**Figure S13.** The thermal stability of the control and target devices. The error bars represent the standard deviation for 10 devices.


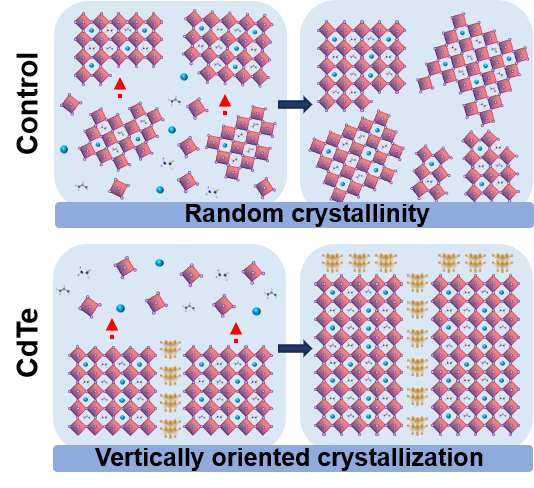


**Figure S14.** Schematic illustration of crystallization process of different perovskite films.

**References**

[1] L. Ye, J. Wu, S. Catalán-Gómez, L. Yuan, R. Sun, R. Chen, Z. Liu, J. M. Ulloa, A. Hierro, P. Guo, Y. Zhou, H. Wang, Superoxide radical derived metal-free Spiro-OMeTAD for highly stable perovskite solar cells, *Nat. Commun.* **2024**, *15*, 7889.

[2] G. Kresse, D. Joubert, From ultrasoft pseudopotentials to the projector augmented-wave method, *Phys. Rev. B* **1999**, *59*, 1758.

[3] J. P. Perdew, K. Burke, M. Ernzerhof, Generalized gradient approximation made simple, *Phys. Rev. Lett.* **1996**, *77*, 3865.

[4] S. Grimme, J. Antony, S. Ehrlich, H. Krieg, A consistent and accurate ab initio parametrization of density functional dispersion correction (DFT-D) for the 94 elements H-Pu, *J. Chem. Phys.* **2010**, *132*, 154104.

[5] K. Momma, F. Izumi, VESTA 3 for three-dimensional visualization of crystal, volumetric and morphology data, *J. Appl. Cryst.* **2011**, *44*, 1272.

[6] H. Li, W. Lu, B. Song, J. Zhou, G. Zhao, G. Han, The design of Mn^2+^&Co^2+^ co-doped CdTe quantum dot sensitized solar cells with much higher efficiency, *RSC Adv.* **2020**, *10*, 35701.

[7] T. Xiao, M. Hao, T. Duan, Y. Li, Y. Zhang, P. Guo, Y. Zhou, Elimination of grain surface concavities for improved perovskite thin-film interfaces, *Nat. Energy* **2024**, *9*, 999.

[8] T. Niu, J. Lu, R. Munir, J. Li, D. Barrit, X. Zhang, H. Hu, Z. Yang, A. Amassian, K. Zhao, S. Liu, Stable high-performance perovskite solar cells via grain boundary passivation, *Adv. Mater.* **2018**, *30*, 1706576.

[9] P. Guo, C. Liu, X. Li, Z. Chen, H. Zhu, L. Zhu, X. Zhang, W. Zhao, N. Jia, Q. Ye, X. Xu, R. Chen, Z. Liu, X. Fan, C. Zhi, H. Wang, Laser manufactured nano-MXenes with tailored halogen terminations enable interfacial ionic stabilization of high performance perovskite solar cells, *Adv. Energy Mater.* **2022**, *12*, 2202395.

[10] L. Ye, P. Guo, J. Su, K. Zhang, C. Liu, P. Yang, W. Zhao, P. Zhao, Z. Liu, J. Chang, Q. Ye, H. Wang, Managing secondary phase lead iodide in hybrid perovskites via surface reconstruction for high-performance perovskite solar cells with robust environmental stability, *Angew. Chem. Int. Ed.* **2023**, *62*, e202300678.

[11] N. Wu, T. Yang, Z. Wang, Y. Wu, Y. Wang, C. Ma, H. Li, Y. Du, D. Zhao, S. Wang, P. Liu, W. Huang, X. Ren, S. Liu, K. Zhao, Stabilizing precursor solution and controlling crystallization kinetics simultaneously for high-performance perovskite solar cells, *Adv. Mater.* **2023**, *35*, 2304809.

[12] Z. Lan, H. Huang, S. Du, Y. Lu, C. Sun, Y. Yang, Q. Zhang, Y. Suo, S. Qu, M. Wang, X. Wang, L. Yan, P. Cui, Z. Zhao, M. Li, Cascade reaction in organic hole transport layer enables efficient perovskite solar cells, *Angew. Chem. Int. Ed.* **2024**, *63*, e202402840.

[13] W. Zhao, P. Guo, J. Su, Z. Fang, N. Jia, C. Liu, L. Ye, Q. Ye, J. Chang, H. Wang, Synchronous passivation of defects with low formation energies via terdentate anchoring enabling high performance perovskite solar cells with efficiency over 24%, *Adv. Funct. Mater.* **2022**, *32*, 2200534.

[14] H. Huang, P. Cui, Y. Chen, L. Yan, X. Yue, S. Qu, X. Wang, S. Du, B. Liu, Q. Zhang, Z. Lan, Y. Yang, J. Ji, X. Zhao, Y. Li, X. Wang, X. Ding, M. Li, 24.8%-efficient planar perovskite solar cells via ligand-engineered TiO_2_ deposition, *Joule* **2022**, *6*, 2186.

[15] W. Zhao, P. Guo, J. Wu, D. Lin, N. Jia, Z. Fang, C. Liu, Q. Ye, J. Zou, Y. Zhou, H. Wang, TiO_2_ electron transport layer with p-n homojunctions for efficient and stable perovskite solar cells, *Nano-Micro Lett.* **2024**, *16*, 191.

[16] Z. Chen, Y. Li, Z. Liu, J. Shi, B. Yu, S. Tan, Y. Cui, C. Tan, F. Tian, H. Wu, Y. Luo, D. Li, Q. Meng, Reconfiguration toward self-assembled monolayer passivation for high-performance perovskite solar cells, *Adv. Energy Mater.* **2023**, *13*, 2202799.

[17] W. Zhao, D. Lin, P. Guo, N. Jia, J. Wu, Q. Ye, F. Yan, H. Wang, Omnibearing molecular-locking of perovskite lattice enables high-performance perovskite solar cells with Efficiency over 26%, *Adv. Funct. Mater.* **2024**, 2423096.

[18] X. Ma, X. Yang, M. Wang, R. Qin, D. Xu, C. Lan, K. Zhao, Z. Liu, B. Yu, J. Gou, S. F. Liu, Comprehensive passivation on different charged ions and defects for high efficiency and stable perovskite solar cells, *Adv. Energy Mater.* **2024**, 2402814.

[19] S. Qu, H. Huang, J. Wang, P. Cui, Y. Li, M. Wang, L. Li, F. Yang, C. Sun, Q. Zhang, P. Zhu, Y. Wang, M. Li, Revealing and inhibiting the facet-related ion migration for efficient and stable perovskite solar cells, *Angew. Chem. Int. Ed.* **2024**, e202415949.

[20] S. Du, H. Huang, Z. Lan, P. Cui, L. Li, M. Wang, S. Qu, L. Yan, C. Sun, Y. Yang, X. Wang, M. Li, Inhibiting perovskite decomposition by a creeper-inspired strategy enables efficient and stable perovskite solar cells, *Nat. Commun.* **2024**, *15*, 5223.

[21] L. Yan, H. Huang, P. Cui, S. Du, Z. Lan, Y. Yang, S. Qu, X. Wang, Q. Zhang, B. Liu, X. Yue, X. Zhao, Y. Li, H. Li, J. Ji, M. Li, Fabrication of perovskite solar cells in ambient air by blocking perovskite hydration with guanabenz acetate salt, *Nat. Energy* **2023**, *8*, 1158.

[22] X. Wang, H. Huang, M. Wang, Z. Lan, P. Cui, S. Du, Y. Yang, L. Yan, Q. Zhang, S. Qu, M. Li, Oriented molecular bridge constructs homogeneous buried interface for perovskite solar cells with efficiency over 25.3%, *Adv. Mater.* **2024**, *36*, 2310710.

[23] Q. Zhang, H. Huang, Y. Yang, M. Wang, S. Qu, Z. Lan, T. Jiang, Z. Wang, S. Du, Y. Lu, Y. Suo, P. Cui, M. Li, A universal ternary solvent system of surface passivator enables perovskite solar cells with efficiency exceeding 26%, *Adv. Mater.* **2024**, 2410390.

[24] D. He, D. Ma, J. Zhang, Y. Yang, J. Ding, C. Liu, X. Liu, Y. Yu, T. Liu, C. Chen, M. Li, J. Chen, Universal ion migration suppression strategy based on supramolecular host-guest interaction for high-performance perovskite solar cells, *Adv. Mater.* **2025**, 2505115.

[25] X. Deng, F. Qi, F. Li, S. Wu, F. R. Lin, Z. Zhang, Z. Guan, Z. Yang, C.-S. Lee, A. K. Y. Jen, Co-assembled monolayers as hole-selective contact for high-performance inverted perovskite solar cells with optimized recombination loss and long-term stability, *Angew. Chem. Int. Ed.* **2022**, *61*, e202203088.

[26] Y. Li, Y. Duan, Z. Liu, L. Yang, H. Li, Q. Fan, H. Zhou, Y. Sun, M. Wu, X. Ren, N. Yuan, J. Ding, S. Yang, S. Liu, In situ synthesized low-dimensional perovskite for >25% efficiency stable MA-free perovskite solar cells, *Adv. Mater.* **2024**, *36*, 2310711.

[27] Y. Li, Y. Duan, J. Feng, Y. Sun, K. Wang, H. Li, H. Wang, Z. Zang, H. Zhou, D. Xu, M. Wu, Y. Li, Z. Xie, Z. Liu, J. Huang, Y. Yao, Q. Peng, Q. Fan, N. Yuan, J. Ding, S. Liu, Z. Liu, 25.71 %-efficiency FACsPbI_3_ perovskite solar cells enabled by a thiourea-based isomer, *Angew. Chem. Int. Ed.* **2024**, *63*, e202410378.

[28] W. Peng, K. Mao, F. Cai, H. Meng, Z. Zhu, T. Li, S. Yuan, Z. Xu, X. Feng, J. Xu, M. D. McGehee, J. Xu, Reducing nonradiative recombination in perovskite solar cells with a porous insulator contact, *Science* **2023**, *379*, 683.

[29] G. Li, Z. Su, L. Canil, D. Hughes, M. H. Aldamasy, J. Dagar, S. Trofimov, L. Wang, W. Zuo, J. J. Jerónimo-Rendon, M. M. Byranvand, C. Wang, R. Zhu, Z. Zhang, F. Yang, G. Nasti, B. Naydenov, W. C. Tsoi, Z. Li, X. Gao, Z. Wang, Y. Jia, E. Unger, M. Saliba, M. Li, A. Abate, Highly efficient p-i-n perovskite solar cells that endure temperature variations, *Science* **2023**, *379*, 399.

[30] Z. Zhu, K. Mao, K. Zhang, W. Peng, J. Zhang, H. Meng, S. Cheng, T. Li, H. Lin, Q. Chen, X. Wu, J. Xu, Correlating the perovskite/polymer multi-mode reactions with deep-level traps in perovskite solar cells, *Joule* **2022**, *6*. 2849.

[31] S. M. Park, M. Wei, J. Xu, H. R. Atapattu, F. T. Eickemeyer, K. Darabi, L. Grater, Y. Yang, C. Liu, S. Teale, B. Chen, H. Chen, T. Wang, L. Zeng, A. Maxwell, Z. Wang, K. R. Rao, Z. Cai, S. M. Zakeeruddin, J. T. Pham, C. M. Risko, A. Amassian, M. G. Kanatzidis, K. R. Graham, M. Grätzel, E. H. Sargent, Engineering ligand reactivity enables high-temperature operation of stable perovskite solar cells, *Science* **2023**, *381*, 209.

[32] F. Li, X. Deng, Z. Shi, S. Wu, Z. Zeng, D. Wang, Y. Li, F. Qi, Z. Zhang, Z. Yang, S.-H. Jang, F. R. Lin, S. W. Tsang, X.-K. Chen, A. K. Y. Jen, Hydrogen-bond-bridged intermediate for perovskite solar cells with enhanced efficiency and stability, *Nat. Photonics.* **2023**, *17*, 478.

[33] S. Zhang, F. Ye, X. Wang, R. Chen, H. Zhang, L. Zhan, X. Jiang, Y. Li, X. Ji, S. Liu, M. Yu, F. Yu, Y. Zhang, R. Wu, Z. Liu, Z. Ning, D. Neher, L. Han, Y. Lin, H. Tian, W. Chen, M. Stolterfoht, L. Zhang, W.-H. Zhu, Y. Wu, Minimizing buried interfacial defects for efficient inverted perovskite solar cells, *Science* **2023**, *380*, 404.

[34] Q. Tan, Z. Li, G. Luo, X. Zhang, B. Che, G. Chen, H. Gao, D. He, G. Ma, J. Wang, J. Xiu, H. Yi, T. Chen, Z. He, Inverted perovskite solar cells using dimethylacridine-based dopants, *Nature* **2023**, *620*, 545.

[35] F. Ye, S. Zhang, J. Warby, J. Wu, E. Gutierrez-Partida, F. Lang, S. Shah, E. Saglamkaya, B. Sun, F. Zu, S. Shoaee, H. Wang, B. Stiller, D. Neher, W.-H. Zhu, M. Stolterfoht, Y. Wu, Overcoming C60-induced interfacial recombination in inverted perovskite solar cells by electron-transporting carborane, *Nat. Commun.* **2022**, *13*, 7454.

[36] J.-H. Kim, Y. R. Kim, J. Kim, C.-M. Oh, I.-W. Hwang, J. Kim, S. Zeiske, T. Ki, S. Kwon, H. Kim, A. Armin, H. Suh, K. Lee, Efficient and stable perovskite solar cells with a high open-circuit voltage over 1.2 V achieved by a dual-side passivation layer, *Adv. Mater.* **2023**, *35*, 2300754.

[37] W. Peng, Y. Zhang, X. Zhou, J. Wu, D. Wang, G. Qu, J. Zeng, Y. Xu, B. Jiang, P. Zhu, Y. Du, Z. Li, X. Lei, Z. Liu, L. Yan, X. Wang, B. Xu, A versatile energy-level-tunable hole-transport layer for multi-composition inverted perovskite solar cells, *Energy Environ. Sci.* **2025**, *18*, 874.

[38] J. J. Yoo, G. Seo, M. R. Chua, T. G. Park, Y. Lu, F. Rotermund, Y.-K. Kim, C. S. Moon, N. J. Jeon, J.-P. Correa-Baena, V. Bulović, S. S. Shin, M. G. Bawendi, J. Seo, Efficient perovskite solar cells via improved carrier management, *Nature* **2021**, *590*, 587.
