# Supplementary material for: CdTe Quantum Dots Encapsulated on Perovskite Grains Enable Highly Efficient and Stable Perovskite Solar Cells
Source: Adv Mater. 2026 Jan 10;38(12):e21104. doi: 10.1002/adma.202521104 (PMC12933008; doi:10.1002/adma.202521104)
Supplement: Supplementary file 2 — Supporting file 2: adma72124‐sup‐0002‐DataFile.zip. [file ADMA-38-e21104-s002.zip › Certified PCE.pdf]

中国认可
国际互认
检测
CALIBRATION
CNAS L0641

210220110107

检 测 报 告

Test Report

报告编号：
Report No. JLY20250517W

样 品 名 称<br>Sample Name	Perovskite solar cell
委 托 单 位<br>Name of Client	西北工业大学<br>Northwestern Polytechnical University
生产单位<br>Manufacturer	西北工业大学

天津市计量监督检测科学研究院
Tianjin Institute of Metrological Supervision and Testing

天津市计量监督检测科学研究院
检验检测专用章
1201040230084

注　意　事　项

Attention

1. 检测报告和骑缝处未加盖“检验检测专用章”无效。
   The report is invalid without test report special stamp in report and the riding seam.

2. 检测只对来样及当时状态负责。
   The report just be responsible for the sample and Status at the time.

3. 检测报告无编制、审核、批准人员签字无效
   The report is invalid without signature of the compile, censor and approver.

4. 检测报告涂改无效。
   The report is invalid if it be altered.

5. 部分复制报告无效。
   Invalid partial copy report

6. 对检测结论若有异议，应于收到检测报告之日起15日内向检测单位提出，逾期不予受理。
   Any objection concerning the report should be submitted to the institute in 15 days after receiving the report. Any request would be refused if it is overdue.

本院地址1：天津市南开区科研西路4号
Add1.: No.4 KeYan West Road, NanKai District, Tianjin, China
本院地址2：天津市宝坻区节能环保工业区内中关村大道北侧
Add2.:North of Zhongguancun Avenue, Energy-Saving and Environmental Protection Industrial Zone, Baodi District, Tianjin, China
本院地址3：天津市河西区解放南路449号
Add3.No.449, Jiefang South Road, Hexi District, Tianjin, China
本院地址4：天津市河西区五号堤路18号
Add4.No.18, Dilu, No.5, Hexi District, Tianjin, China

服务电话：022-23009329
Telephone
传真号码：022-23009354
Fax
投诉电话：022-23009322
Complaint Tel.
邮政编码：300192
Post Code
电子邮件：timstbmd@126.com
E-mail

天津市计量监督检测科学研究院

Tianjin Institute of Metrological Supervision and Testing

检测报告

Test Report

报告编号：JLY20250517W
Report No.

样品名称<br>Sample Name	Perovskite solar cell				
规格型号<br>Specification Type	/	商标/出厂编号<br>Brand/Serial Number	/<br>6-1-1	生产日期/批号<br>Produce Date/ Serial Number	2025-03-19
样品等级<br>Sample Grade	/	样品描述<br>Sample Description	外观良好，无明显可见缺陷	样品接收时间<br>Sample receive time	2025-03-19
委托日期<br>Delivery Date	2025-03-19	送样人员<br>Delivered by	郭鹏飞	样品数量<br>Sample Quantity	1块
委托单位名称及联系电话<br>Name of Client	西北工业大学<br>Northwestern Polytechnical University/18700926052				
生产单位名称<br>Manufacturer	西北工业大学				
检测时间<br>Test Time	2025-03-19	检测地点<br>Test Location	本院五号堤路院区光伏产业计量部106室	检测环境<br>Test Condition	温度：24.9℃<br>相对湿度：31.5%
检测依据<br>Test Standard	IEC 60904-1:2020《光伏器件 第1部分：光伏电流-电压特性的测量》				
检测结论<br>Test Conclusion	/<br>（检验检测专用章）<br>（Stamp of Test Institution）<br>签发日期：2025-06-23<br>Issue Date: Y M D				
备注<br>Remark	/				

编制：
Compiled by

审核：
Checked by

批准：
Approved by

天津市计量监督检测科学研究院

Tianjin Institute of Metrological Supervision and Testing

检测报告

Test Report

检 测 仪 器 设 备

序号<br>No.	仪器设备名称<br>Instrument	型号/规格<br>Type	设备编号<br>Instrument No.	证书有效期<br>Expiry Date	证书编号<br>Certificate No.
1	电池片 I-V 测试系统<br>I-V measurement system for solar cell	VS-6831S	21VJ002	2025.08.16	CGFg124070291

天津市计量监督检测科学研究院

Tianjin Institute of Metrological Supervision and Testing

检测报告

Test Report

报告编号：JLY20250517W　　　　　　　　　　共 4 页　第 3 页

Report No.　　　　　　　　　　　　　　　Page No.4-3

受 检 样 品 信 息

样品编号<br>Sample Number	规格型号<br>Sample Type	产品编号<br>Product No.	产品序列号<br>Serial Number
20250517-1	—	6-1-1	—

天津市计量监督检测科学研究院

Tianjin Institute of Metrological Supervision and Testing

检测报告

Test Report

报告编号：JLY20250517W　　　　共 4 页　　第 4 页

Report No.　　　　Page No.4-4

检 测 项 目 及 结 果

测试时间<br>Date	2025.03.19							
测试条件<br>Condition	使用稳态 AAA 级太阳模拟器，在 AM1.5G，1000W/m^2，25.0℃条件下测试<br>Sample was tested under the condition of AM1.5G, 1000W/m^2, 25.0℃ with a steady-state class calibrated AAA solar simulator							
有效面积<br>Active area	0.069316cm^2<br>备注：器件的有效面积是由带固定孔径的薄金属掩模板量化。<br>Remark: Designated area defined by thin metal aperture mask.							
样品编号<br>№	V_{oc}<br>(V)	I_{sc}<br>(mA)	J_{sc}<br>(mA/cm^2)	P_{max}<br>(mW)	V_{Pmax}<br>(V)	I_{Pmax}<br>(mA)	FF<br>(%)	η<br>(%)
20250517-1	1.205	1.820	26.25	1.804	1.040	1.734	82.27	26.02
测试程序<br>Test program settings	起始电压：<br>Starting voltage:	+1.22V						
	终止电压：<br>Termination voltage:	-0.10V						
	扫描间隔：<br>Scan interval:	0.02V						
	延迟时间：<br>Delay time:	0.01s						
I-V 曲线图<br>*I-V* curve	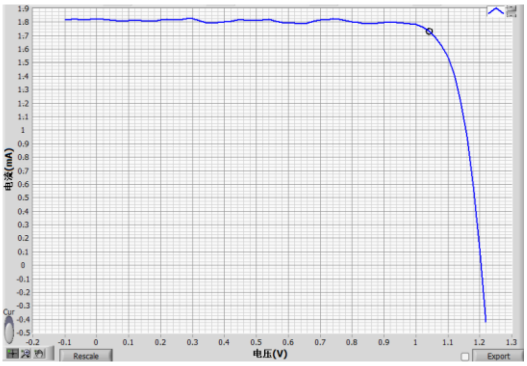							
备注<br>Remark	—							

————以下空白————

Blank below
